# Laser speckle rheological microscopy reveals wideband viscoelastic spectra of biological tissues

**DOI:** 10.1126/sciadv.adl1586

**Published:** 2024-05-08

**Authors:** Nichaluk Leartprapun, Ziqian Zeng, Zeinab Hajjarian, Veerle Bossuyt, Seemantini K. Nadkarni

**Affiliations:** ^1^ Wellman Center for Photomedicine, Massachusetts General Hospital, Harvard Medical School, Boston, MA 02114, USA.; ^2^ Department of Pathology, Massachusetts General Hospital, Boston, MA 02114, USA.

## Abstract

Viscoelastic transformation of tissue drives aberrant cellular functions and is an early biomarker of disease pathogenesis. Tissues scale a range of viscoelastic moduli, from biofluids to bone. Moreover, viscoelastic behavior is governed by the frequency at which tissue is probed, yielding distinct viscous and elastic responses modulated over a wide frequency band. Existing tools do not quantify wideband viscoelastic spectra in tissues, leaving a vast knowledge gap. We present wideband laser speckle rheological microscopy (WB-SHEAR) that reveals elastic and viscous response over sub-megahertz frequencies previously not investigated in tissue. WB-SHEAR uses an optical, noncontact approach to quantify wideband viscoelastic spectra in specimens spanning a range of moduli from low-viscosity fibrin to highly elastic bone. Via laser scanning, micromechanical imaging is enabled to access wideband viscoelastic spectra in heterogeneous tumor specimens with high spatial resolution (25 micrometers). The ability to interrogate the viscoelastic landscape of diverse biospecimens could transform our understanding of mechanobiological processes in various diseases.

## Teaser

Microscopy technique, WB-SHEAR, enables “viscoelastic fingerprinting” in biomimetic scaffolds and biological tissues.

## INTRODUCTION

The evidence that mechanical factors are decisive participants in disease pathogenesis is unequivocal (*1*–*3*). Aberrant mechanical remodeling is implicated in a broad spectrum of pathologies including the onset and progression of neoplasms (*4*–*6*), hematological disorders (*7*, *8*), cardiovascular diseases (*9*–*11*), fibro-proliferative disorders (*12*), and several orthopedic conditions (*13*). For instance, alterations in tissue stiffness, a frequent consequence of desmoplastic reaction, have been linked to malignancy and chemoresistance in solid tumors (*14*–*18*). Compromised bone strength is associated with diminished bone density and may portend a higher risk of osteoporotic fractures (*19*, *20*). Coagulopathy is associated with modulation in the mechanical properties and stability of blood clots (*21*), motivating the development of mechanics-based point-of-care diagnostic devices for hemostasis management (*22*).

Biological tissues scale a broad range of mechanical moduli, from low-viscosity biofluids to stiff bone structures. Current insights have largely relied on a single mechanical descriptor of elasticity. However, tissue is composed of a complex network of cells and fibrillar proteins, surrounded by interstitial fluid containing 90% water. As a result, the intricate structure and composition of tissue yield both elastic (*G*′) and viscous (*G*″) behaviors that together contribute to the complex shear (viscoelastic) modulus, G*(ω) = *G*′(ω) + *iG*″(ω), modulated over a wide range of temporal angular frequency, ω*,* at which the tissue is interrogated. Living cells sense both elastic and viscous mechanical cues at varying ω frequencies depending on the timescales at which they probe their microenvironment (*23*–*25*). Cells respond to viscous dissipation in the extracellular matrix (ECM) via intracellular signaling in ways not explained by changes in elasticity alone (*26*–*30*). For instance, in vitro cell culture studies show that cancer cell invasion is simultaneously accompanied by two seemingly opposing mechanical transformations in the ECM: stiffening and “liquidization,” each dominating at different frequency regimes (*31*). Thus, a single metric of elasticity or viscoelasticity over a small frequency band only provides a limited snapshot, inadequate for capturing the full complexity of mechanical cues that regulate mechanobiological processes. However, viscoelastic behavior over a wide band of frequencies have yet to be investigated in whole (intact) tissue.

Existing techniques do not support frequency-dependent measurement of elastic and viscous moduli up to the megahertz regime in biological tissues (*32*). Conventional rheometry can only provide bulk measurement at very low frequencies, typically over a few hertz (*33*). Although conventional microrheology techniques—based on particle tracking (*34*), dynamic light scattering (DLS) (*35*, *36*), diffusing wave spectroscopy (DWS) (*37*), and optical manipulation (*38*–*40*)—are capable of measurement over a wide frequency range, they are not applicable in whole tissue due to the reliance on exogenous probe particles. Among whole-tissue techniques, optical coherence elastography typically provides quasi-static measurement of elasticity or dynamic measurement over limited frequencies (typically <10 kHz) (*41*, *42*). Meanwhile, Brillouin microscopy provides longitudinal modulus in the gigahertz range at high spatial resolution, but the interpretation of this modulus in relation to the widely referenced Young’s or shear modulus remains a challenge (*43*). Thus, there is a vast knowledge gap in the kilohertz to megahertz range in which the mechanical behavior of whole tissue remains largely unknown. Linked to fundamental biophysical processes, study in this regime has the potential to unlock previously unidentified sources of micromechanical contrast for improved disease prognostication, as well as generate unique insights in the field of mechanobiology that has thus far relied on a single elastic modulus at a low frequency. Together, there are no existing tools to comprehensively assess tissue viscoelasticity over a broad range of mechanical moduli and across a wide band of frequencies relevant for studying mechanobiological processes in various pathologies.

We present wideband laser speckle rheological microscopy (WB-SHEAR) to measure frequency-dependent elastic and viscous moduli in biological tissues up to the sub-megahertz regime. On the basis of the fluctuation of speckle—an interference pattern formed upon laser illumination of tissue—induced by natural thermal motion of native tissue structures, SHEAR provides passive microrheological measurement in an all-optical, noninvasive, and noncontact manner (*44*–*47*). We reveal wideband viscoelastic spectra over frequencies from ω ~ 1 to 10^5^ rad/s in various biospecimens that cover a broad range of shear moduli from ~0.1 to 10^7^ Pa, including fibrin scaffolds, whole-blood clots, breast tumors, and bone. Furthermore, by conducting two-dimensional (2D) scanning, we demonstrate that spatially variant, wideband viscoelastic spectra can be mapped with a high spatial resolution (spatial sampling at 25 μm) to capture heterogeneous micromechanical properties of distinct tissue composites within human tumor specimens. WB-SHEAR provides the only tool to measure the full complex shear modulus, permitting the distinction of elastic and viscous contributions to the overall mechanical behavior, over 5 decades of frequencies in biological tissues that span over 7 decades in shear moduli. By enabling the investigation of tissue mechanical properties over previously inaccessible frequency regimes, WB-SHEAR reveals never-before-seen distinct frequency-dependent viscoelastic signatures of biological tissues that exist up to the sub-megahertz regimes.

## RESULTS

### Principle of WB-SHEAR

Biological tissues are composed of numerous microscopic light scattering structures that are susceptible to naturally occurring thermal (Brownian) displacements, the magnitude and frequencies of which are governed by the local viscoelastic behavior of the microenvironment. Consequently, scattering structures in softer cellular or adipose tissues may exhibit larger and more rapid displacements compared to stiffer fibrous regions. Upon illumination by a coherent beam, light scattered by these dynamic structures interfere to form a temporally fluctuating speckle pattern. By recording a time series of speckle patterns and analyzing its magnitude and rate of fluctuation, the frequency-dependent viscoelastic behavior of the material can be reconstructed. On the basis of this principle, we developed SHEAR to map |G*| with high spatial resolution in various biological specimens, including hydrogels (*47*), blood (*48*–*50*), atherosclerotic plaques (*51*, *52*), and breast tumors (*53*). Unlike traditional DLS and DWS approaches, SHEAR is not restricted to either transparent diluted samples (for DLS) or highly concentrated turbid suspensions (for DWS) that require incorporating specific amount of exogenous scattering particles. Rather, it directly exploits displacements of native scatterers that are already present in the sample and navigates a range of intrinsic tissue scattering properties that lies between the two scattering limits. Nevertheless, our previous implementation of SHEAR was limited to the measurement of shear modulus magnitude at low frequencies over tens of hertz and, thus, did not provide a complete picture of the complex wideband frequency–dependent elastic and viscous behavior of tissue. The WB-SHEAR approach detailed here addresses this critical limitation and enables the distinct measurements of frequency-dependent shear storage, *G*′(ω), and loss, *G*″(ω), moduli to distinguish the contributions of various tissue constituents exhibiting elastic and viscous behaviors in tissue, spanning sub-hertz to sub-megahertz frequency range.

**Fig. 1.**
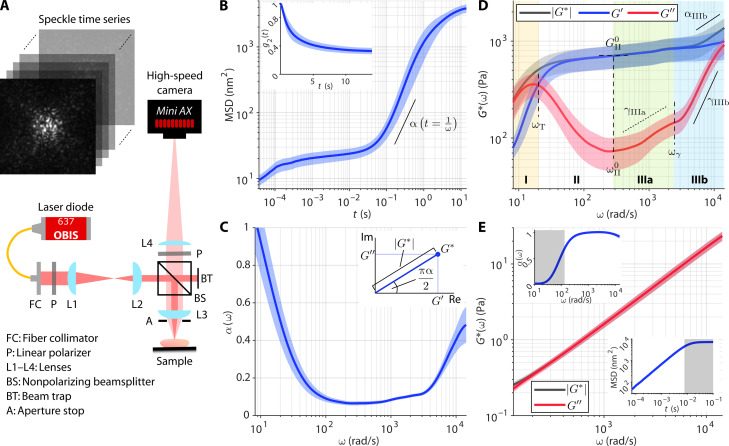
Measurement of wideband viscoelastic spectra in fibrin constructs. (**A**) Optical setup of the WB-SHEAR system. (**B**) Time-dependent MSD of polymerized fibrin construct computed from the intensity autocorrelation (inset) of the acquired speckle time series. (**C**) Frequency-dependent α(ω) obtained from the log-log derivative of the MSD in (C). Inset illustrates the role of α in determining the relative contribution of *G*′ and *G*″ in the complex modulus, G* = *G*′ + *iG*″. (**D**) Frequency-dependent |G*(ω)| (black), *G*′(ω) (blue), and *G*″(ω) (red) of fibrin construct computed from MSD and α in (B) and (C). (**E**) |G*(ω)| (black) and *G*″(ω) (red) of pure fibrinogen solution containing 3-μm-diameter polystyrene particles, showing linearly increasing G*(ω) = *G*″(ω) indicated by a log-log slope of 1. Insets show the measured MSD and α; shaded areas indicate timescales over which the speckle pattern has completely decorrelated because of the fast dynamics in aqueous solution. In (B) to (E), solid curve and shaded area represent means ± SD of *N* = 9 technical replicates (three independent locations in each of three biological replicates), respectively. [In (D), ω_T_ = 20 ± 3 rad/s, ω^0^_II_ = 300 ± 100 rad/s, *G*^0^_II_ = 700 ± 200 Pa, γ_IIIa_ = 0.4 ± 0.1, ω_γ_ = 2420 ± 70 rad/s, γ_IIIb_ = 1.2 ± 0.1, α_IIIb_ = 0.50 ± 0.09. For α and γ, dotted and solid lines indicate that power scaling exponents are <0.5 and ≥0.5, respectively. Values of all annotated spectroscopic parameters are tabulated in table S1.]

The highest frequency accessible by WB-SHEAR is physically limited by the speckle acquisition frame rate, which directly determines the smallest timescale at which the rate of speckle fluctuation can be measured. We use a high-speed complementary metal-oxide semiconductor (CMOS) camera (Photron, Mini AX200 type 900k) that can support a frame rate up to 540 kHz with a sensor bit depth of 12 bits and region of interest (ROI) of 128 × 32 pixels, corresponding to a field of view of 192 μm by 42 μm (1.5 μm per pixel). The optical system is designed to achieve a camera pixel-to-speckle ratio of 3.5 pixels/speckles (along one dimension), ensuring sufficient spatial sampling to capture fully developed speckle while also maximizing the number of individual speckles captured within the diffused reflectance profile (DRP) of the focused illumination. Speckle time series is acquired in a 180° backscattered configuration at perpendicular polarization to the illumination (Fig. 1A; see Materials and Methods for detailed description of the optical system and speckle acquisition procedure). Speckle fluctuation is evaluated by computing the ensemble-averaged intensity autocorrelation function, *g*_2_(*t*), from which the time-dependent mean square displacement (MSD) of the scatterers is obtained according to Eqs. 1 and 2 in Materials and Methods (*44*, *45*, *47*). Given the focused illumination, we computed the ensemble average over a circular analysis ROI around the illumination center, with diameter ranging from 36 to 75 μm depending on the spatial extent of the DRP (see Materials and Methods). Notably, Eq. 2 is an empirical approximation of the DWS formulation that allows SHEAR to account for an arbitrary set of optical properties, estimated via diffusion theory from the radial profile of the DRP in each sample (fig. S1; further details in Materials and Methods), as previously described (*44*, *45*). An example of *g*_2_(*t*) curve and time-dependent MSD measured in a fibrin hydrogel are shown in Fig. 1B.

Under thermal equilibrium, the frequency-dependent shear viscoelastic moduli can be derived from the measured MSD via the generalized Stokes-Einstein relation (GSER). To reconstruct both the elastic, *G*′(ω), and viscous, *G*″(ω), moduli, the log-log derivative of the MSD with respect to time (slope annotated on Fig. 1B) is computed to obtain the frequency-dependent power scaling law, α(ω), where MSD∝*t*^α(ω)^ at *t* = 1/ω (Fig. 1C). While the MSD itself is inversely proportional to the magnitude |G*|, its power scaling law α determines the phase angle ∠G* that governs the relative contribution of the real, *G*′, and imaginary, *G*″, parts of the complex modulus (inset of Fig. 1C; see Eq. 3 in Materials and Methods for the full expression) (*54*–*56*). Here, α = 0 indicates a purely elastic solid-like behavior (i.e., |G*| = *G*′), whereas α = 1 indicates a purely viscous fluid–like behavior (i.e., |G*| = *G*″). Meanwhile, viscoelastic materials exhibit frequency-dependent α(ω) that varies between the two limits (i.e., 0 < α < 1), where α = 0.5 corresponds to *G*′ = *G*″ and deviation in either direction indicates more elasticity-dominant (α < 0.5, *G*′ > *G*″) or viscosity-dominant (α > 0.5, *G*′ < *G*″) behavior. For the fibrin construct, α(ω) decreases from α ~ 1 at ω ≤ 10 rad/s to α ~ 0.1 at 100 ≤ ω < 3000 rad/s, then approaches α ~ 0.5 at ω > 10^4^ rad/s (Fig. 1C), giving rise to the frequency-dependent *G*′(ω) and *G*″(ω) spectra (Fig. 1D). In contrast, a solution of unpolymerized fibrinogen (fibrin precursors) exhibits a linearly increasing MSD and a constant α(ω) = 1 (insets of Fig. 1E), resulting in a purely viscous behavior with |G*| = *G*″, where *G*″ increases linearly with ω as expected of a linear viscous fluid (Fig. 1E).

### Frequency-dependent viscoelastic behavior of fibrous polymer construct

We measured *G*′(ω) and *G*″(ω) of polymerized fibrin constructs to examine the frequency-dependent viscoelastic behavior of a typical fibrous ECM (Fig. 1D). Fibrin was chosen as an example for its clinical relevance in hematology, infection, and wound healing. WB-SHEAR up to ω > 10^4^ rad/s reveals four regimes of distinct viscoelastic behaviors across the frequency spectrum. In regime I, the viscoelastic behavior of the fibrin construct is dominated by viscous contribution with *G*″ > *G*′. The low-frequency fluid-like behavior is attributed to the relaxation dynamics of the hydrogel scaffold (*57*, *58*). As the frequency increases, *G*′ gradually approaches *G*″ until they crossover (i.e., α = 0.5) at the fluid-to-solid transition frequency, ω_T_ = 20 ± 3 rad/s. In regime II, the fibrin construct reaches its most elastic behavior with α < 0.1 (Fig. 1C), as *G*″ decreases to a local minimum at frequency ω^0^_II_ = 300 ± 100 rad/s and *G*′ reaches the “elastic plateau modulus” of *G*^0^_II_ = 700 ± 200 Pa. Governed by the collective dynamics of the fibrin fiber network (*36*, *57*, *58*), the elastic plateau is the regime at which microrheological measurement by WB-SHEAR is in closest agreement with bulk rheometry (fig. S2). Here, the elastic plateau extends for ~2 decades, with both *G*′ and |G*| remaining relatively constant. In regime III, hidden beneath the elastic plateau, *G*″ increases following a power scaling law *G*″∝ω^γ^. Two distinct scaling laws can be observed: first, with the exponent of γ_IIIa_ = 0.4 ± 0.1, then transitions to γ_IIIb_ = 1.2 ± 0.1 at frequency ω_γ_ = 2420 ± 70 rad/s. Within regime IIIb, the increasing contribution of *G*″(ω) causes the overall modulus magnitude to also follow a power scaling law |G*|∝ω^α^ with an exponent of α_IIIb_ = 0.50 ± 0.09. The shift from |G*| plateau to high-frequency power scaling can be interpreted as the transition from the network-level elastic behavior at intermediate frequencies to the single filament–level fluctuation at higher frequencies (*36*, *57*, *58*). The power scaling exponent of α ~ 0.5 is consistent with the bending fluctuation of a Rouse flexible polymer (*57*, *58*).

Values of all spectroscopic parameters annotated in Fig. 1 are summarized in table S1. These wideband spectroscopic parameters together describe the intricate frequency-dependent elastic and viscous behaviors of polymerized fibrin, a fibrillar protein commonly found in biological tissue. The behaviors observed here differ from those of macrorheology in fibrin constructs except at the elastic plateau. Notably, the fluid-to-solid transition between regimes I and II is not observed (i.e., *G*′ > *G*″ from the lowest frequencies) at the macroscale (*59*–*61*). However, previous microrheological studies have revealed this fluid-to-solid transition in low-concentration partially cross-linked hydrogels, where the degree of cross-linking heavily influenced the viscous contribution at low frequencies (*39*, *62*). Furthermore, the power scaling behaviors observed at the higher frequency regimes, beyond the applicable range of conventional rheometry, have been widely observed via microrheology in biofluids and biomimetic scaffolds (*36*, *39*, *62*–*66*). In the following sections, we show that the spectroscopic analysis described above can be applied to a wide range of complex clinical specimens to reveal distinct mechanical signatures in tissues such as whole-blood clots, breast tumors, and bone.

### Wideband viscoelastic spectra of clinical whole-blood clots

**Fig. 2.**
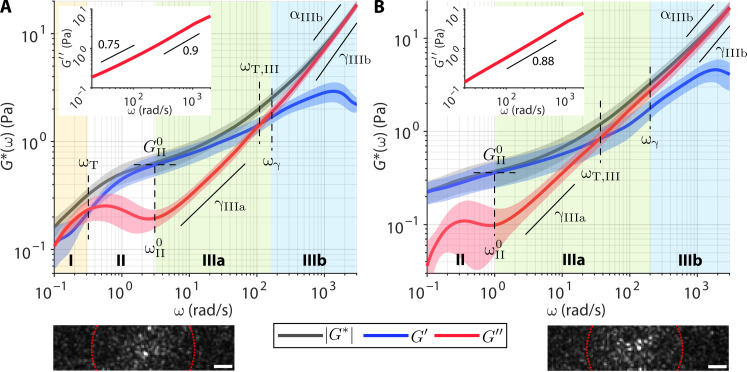
Wideband viscoelastic spectra of clinical blood clots. Frequency-dependent |G*(ω)| (black), *G*′(ω) (blue), and *G*″(ω) (red) of clots formed by whole-blood samples with fibrinogen contents at the (**A**) upper (high-FIB, 5.15 mg/ml) and (**B**) lower (low-FIB, 2.12 mg/ml) limits of the normal range. Solid curve and shaded area represent means ± SD of *N* = 5 technical replicates (five independent locations in one biological replicate). (For α and γ, solid lines indicate that all power scaling exponents are ≥0.5. Values of all annotated spectroscopic parameters are tabulated in table S1.) Insets show *G*″(ω) in pure whole blood before clot initiation. Annotated numbers indicate the power scaling law of *G*″(ω). Images below show a frame from the speckle time series, with the analysis ROI indicated by red dotted line. Scale bar, 20 μm.

Blood clots are composed of polymerized fibrin and other cellular components such as platelets and red blood cells (RBCs) tightly held within the fibrous scaffolds. Mechanical properties of blood clots have emerged as an important player in the management of thrombotic and bleeding complications in the clinical settings (*49*, *50*). We obtained two clinical blood samples from the Massachusetts General Hospital (MGH) Core Laboratory [MGH Institutional Review Board (IRB) no. 2017P000419], each presenting high (hereafter “high-FIB”) and low (hereafter “low-FIB”) fibrinogen content associated with potential thrombotic and bleeding risks, respectively. WB-SHEAR was performed in whole blood before (Fig. 2, insets) and after (Fig. 2) clot initiation by kaolin and CaCl_2_. Values of all spectroscopic parameters annotated in Fig. 2 are summarized in table S1.

The viscoelastic behavior of high-FIB clot is similar to that of the purified fibrin construct in regimes I and II, but with both the fluid-to-solid transition and the elastic plateau occurring at lower frequencies of ω_T_ = 0.36 ± 0.09 rad/s and ω^0^_II_ = 3.1 ± 0.7 rad/s, respectively (Fig. 2A). Furthermore, the elastic plateau modulus is lower at *G*^0^_II_ = 0.6 ± 0.2 Pa and extends for less than a decade, indicating lower network elasticity and an overall less solid-like behavior compared to the purified fibrin in Fig. 1D. In regime III, *G*″(ω) power scaling has the exponent of γ_IIIa_ = 0.6 ± 0.2 that transitions to γ_IIIb_ = 0.83 ± 0.01 at ω_γ_ = 160 ± 30 rad/s, suggesting that the viscous behavior approaches that of a purely viscous fluid with γ → 1 (i.e., *G*″ increases linearly with ω) at higher frequencies. A unique behavior in whole-blood clots compared to purified fibrin is the second crossover between *G*′ and *G*″ at ω_T,III_ = 110 ± 50 rad/s—this time, a transition back to a more viscosity-dominant behavior with *G*″ exceeding *G*′. With *G*″ dominating the viscoelastic behavior after this transition, |G*(ω)| follows the same power scaling law with exponent α_IIIb_ = γ_IIIb_. In comparison, the low-FIB clot has a lower elastic plateau modulus of *G*^0^_II_ = 0.3 ± 0.2 Pa that occurs at a frequency of ω^0^_II_ = 0.8 ± 0.7 rad/s (Fig. 2B). Furthermore, the solid-to-fluid transition in regime III also occurs at a much lower frequency of ω_T,III_ = 40 ± 30 rad/s compared to high-FIB. Thus, the low-FIB clot not only has lower elasticity (i.e., more compliant) than the high-FIB clot but also exhibits more *G*″-dominant behavior across a wider range of frequencies, likely due to the lower availability of fibrinogen protein to form a stable fibrin network.

Evidently, the viscoelastic spectra of clots formed by whole blood (Fig. 2) are markedly different from those of the purified fibrin construct (Fig. 1D). The discrepancies are not unexpected, despite the similar fibrinogen concentrations in Figs. 1D and 2A. Unlike in the purified fibrin construct, the viscoelastic behavior of whole-blood clots as probed by WB-SHEAR would also contain substantial contributions from the cellular components (~45% of whole-blood volume) such as RBCs and platelets that fill the pore spaces within the fibrin meshwork. We hypothesize that these cellular components (essentially light scattering membranes surrounded on either side by fluids of the plasma and the cytoplasm) contribute to the relatively more viscous behavior and lower moduli values in whole-blood clots compared to purified fibrin construct. Furthermore, the fact that the fibrin network must form around numerous large (relative to the native fibrin pore sizes) structures of blood cells, and in the presence of multiple proteins and factors present in blood plasma, also likely influences the microstructures of the resulting fibrin scaffold compared to the purified case. In particular, in addition to fibrinogen, the amounts of thrombin, calcium, ionic strength, fibronectin, and albumin have been shown to influence the properties of fibrin scaffolds (*67*–*69*). The complexity of whole blood can also be appreciated from the viscosity measurement before clot initiation. Unlike in solution of unpolymerized fibrinogen molecules (Fig. 1E), *G*″ does not increase linearly with frequency (i.e., α = 1) but instead follows a power law with α(ω) in the range of 0.75 to 0.9 (insets of Fig. 2). Both whole-blood samples are also more viscous, with average viscosity of 4.5 ± 0.2 mPa⋅s (high-FIB) and 4.6 ± 0.1 mPa⋅s (low-FIB) at ω > 200 rad/s, compared to the pure fibrinogen solution with viscosity of 1.43 ± 0.06 mPa⋅s.

### Profiling wideband viscoelastic signatures in human breast cancer

**Fig. 3.**
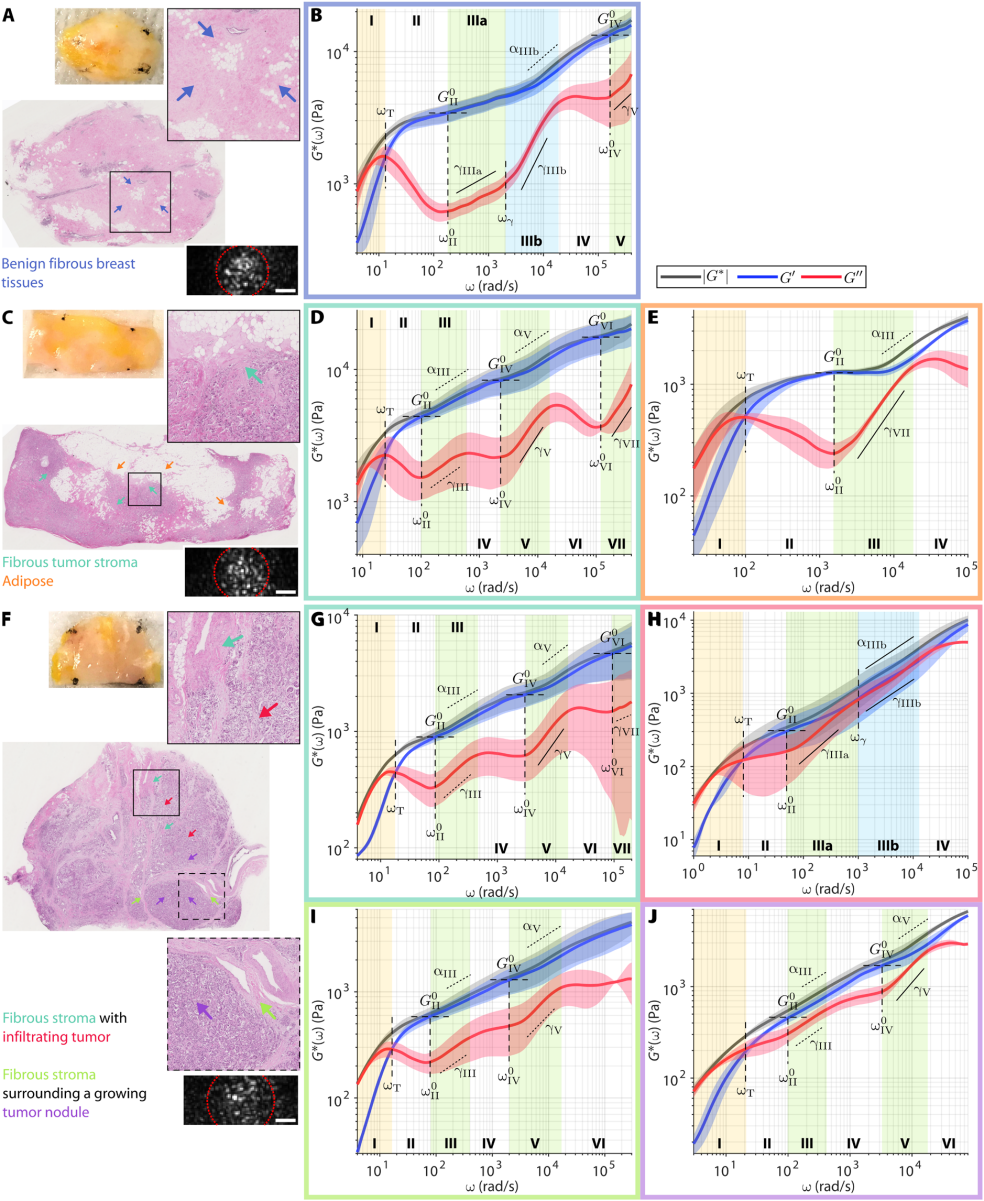
Wideband viscoelastic spectra of the breast tumor microenvironments. Gross photo, H&E slide, example speckle frame (red dotted line indicates analysis ROI; scale bar, 20 μm), and frequency-dependent |G*(ω)| (black), *G*′(ω) (blue), and *G*″(ω) (red) of (**A** and **B**) benign breast tissue, (**C** to **E**) treated invasive carcinoma, and (**F** to **J**) untreated invasive carcinoma. Solid curve and shaded area represent means ± SD of *N* = 3 independent measurement at locations indicated by colored arrows on H&E. Arrows and plot outlines are color-coded for benign fibrous breast tissue (blue), adipose (orange), tumor stroma (dark green and light green), and cellular tumor epithelium (purple and red). (For α and γ, dotted and solid lines indicate that power scaling exponents are <0.5 and ≥0.5, respectively. Values of all annotated spectroscopic parameters are tabulated in table S2.)

Breast tumors are mechanically heterogeneous, both across different molecular subtypes and within a single specimen. We have previously shown that the cellular tumor epithelium tends to have lower |G*| than the fibrous stroma, with tumors of more aggressive molecular subtypes generally exhibiting sharper |G*| gradient across the tumor-stroma interface (*53*). We performed WB-SHEAR on three freshly excised breast specimens from different patients (MGH IRB no. 2011P000301): benign breast tissue from a patient with breast cancer (Fig. 3, A and B), residual tumor after treatment with neoadjuvant chemotherapy (Fig. 3, C to E), and untreated invasive breast carcinoma (Fig. 3, F to J). Across the three specimens, we observe four different types of frequency-dependent viscoelastic behavior corresponding to benign fibrous breast tissue (Fig. 3B), adipose (Fig. 3E), fibrous tumor stroma (Fig. 3, D, G, and I), and cellular tumor epithelium (Fig. 3, H and I). Values of all spectroscopic parameters annotated in Fig. 3 are summarized in table S2.

In benign fibrous breast tissue (Fig. 3A), the wideband viscoelastic spectra are rather reminiscent of that of the purified fibrin constructs in Fig. 1D, except with the moduli magnitude being overall higher (Fig. 3B). Notably, two distinct power scaling laws are also observed for *G*″(ω) in regime III, with both exponents being lower than those of fibrin (γ_IIIa_ = 0.20 ± 0.03, γ_IIIb_ = 0.73 ± 0.05) but transitioning at similar frequency of ω_γ_ = 2000 ± 200 rad/s. Similarly, the increasing contribution of *G*″(ω) in regime IIIb also results in the high-frequency power law behavior of |G*(ω)| with the exponent of α_IIIb_ = 0.33 ± 0.05. Taking the measurement up to ω > 10^5^ rad/s reveals two additional frequency regimes. Regimes IV and V exhibit similar behaviors to regimes II and III, with *G*″ reaching another local minimum at frequency ω^0^_IV_ = 2 × 10^5^ ± 1 × 10^5^ rad/s, accompanied by its own elastic plateau modulus of *G*^0^_IV_ = 13 ± 2 kPa, followed by a power scaling law with exponent of γ_V_ = 0.5 ± 0.1.

Invasive ductal carcinoma after neoadjuvant (presurgical) systemic treatment displays varying levels of cellularity, with interspersed tumor cell clusters within the fibrous tumor stroma and areas of adipose (Fig. 3C). Unlike the benign fibrous breast tissue, the fibrous tumor stroma (green arrows in Fig. 3C) exhibits a characteristic undulation pattern in its viscous behavior (Fig. 3D), where the viscoelastic spectra repeatedly reach a new elastic plateau (regimes II, IV, and VI) followed by a *G*″(ω) power scaling (regimes III, V, and VII), each obeying only a single power law (i.e., γ_IIIb_ in Fig. 3B is not observed). Notably, higher elastic plateau modulus is observed at increasing frequency regimes (*G*^0^_II_ = 4.6 ± 0.9, *G*^0^_IV_ = 8 ± 2, *G*^0^_VI_ = 18 ± 5 kPa), indicating decreased compliance as a function of frequency. The power scaling laws also follow a similar increasing trend for both *G*″ (γ_III_ = 0.33 ± 0.09, γ_V_ = 0.55 ± 0.06, γ_VII_ = 0.6 ± 0.3) and |G*| (α_III_ = 0.22 ± 0.06, α_V_ = 0.26 ± 0.01). An increase in the high-frequency power law exponent may be interpreted as a decrease in the filament flexibility of the fibrillar structures (*57*, *58*). Meanwhile, the viscoelastic spectra of adipose tissue (orange arrows in Fig. 3C) exhibit only one elastic plateau and one *G*″(ω) power scaling regime (Fig. 3E). The elastic plateau occurs at a higher frequency of ω^0^_II_ = 1500 ± 200 rad/s with a lower plateau modulus of *G*^0^_II_ = 1.27 ± 0.06 kPa compared to the fibrous stroma. However, the high-frequency power scaling of both *G*″(ω) and |G*(ω)| is steeper with the exponents of γ_III_ = 0.8 ± 0.1 and α_III_ = 0.44 ± 0.05, respectively.

Compared to the treated sample, the untreated invasive carcinoma displays well-delineated regions of fibrous stroma and cellular tumor epithelium (Fig. 3F). The fibrous stroma with tumor infiltration (dark green arrows in Fig. 3F, top inset) exhibits the characteristic undulation pattern in *G*″ (Fig. 3G) that is similar to that of the stroma in the treated tumor in Fig. 3D. Notably, although the elastic plateau moduli are lower (*G*^0^_II_ = 0.9 ± 0.09, *G*^0^_IV_ = 2.1 ± 0.4, *G*^0^_VI_ = 5 ± 2 kPa), the power scaling exponents for both *G*″(ω) (γ_III_ = 0.44 ± 0.03, γ_V_ = 0.58 ± 0.06, γ_VII_ = 0.35 ± 0.3) and |G*(ω)| (α_III_ = 0.27 ± 0.05, α_V_ = 0.28 ± 0.07) are in the same range as those of the treated tumor. Likewise, the fibrous tumor stroma surrounding a growing nodule of tumor cells (light green arrows in Fig. 3F, bottom inset) also exhibits characteristic undulation in the viscous behavior and power scaling exponents in the same range of α ~ 0.2 to 0.3 (Fig. 3I). In comparison, the cell-dense nodule of tumor (purple arrows in Fig. 3F) exhibits noticeably more viscous behavior, where *G*″ lies close to *G*′ throughout the spectra (Fig. 3J). The higher contribution of *G*″ to the overall modulus is also reflected in the power scaling exponents of |G*(ω)|, with α approaching 0.5 (α_III_ = 0.40 ± 0.01, α_V_ = 0.45 ± 0.07). The viscous behavior is even more dominant in the tumor that is not contained in a nodule but infiltrates the fibrous stroma (red arrows in Fig. 3F), where *G*″ completely overlaps *G*′, resulting in a high frequency |G*(ω)| power scaling law with α_IIIb_ = 0.51 ± 0.09 that extends over a decade (Fig. 3H). The relatively more fluid-like behavior of the infiltrating tumor supports the model of cancer invasion based on cell jamming theory, where the invading tumor appears “liquidized” as it unjams from its solid tumor state (*70*, *71*).

Our results show that the breast tumor microenvironment is an extremely heterogeneous micromechanical landscape, not only in quasi-static elasticity (*72*–*74*) or low-frequency viscoelastic modulus (*53*) but even more remarkably so in the frequency-dependent elastic and viscous behaviors over a wide frequency band, which previously has not been accessible. Notably, although all tissues in Fig. 3 behave similarly at ω < 100 rad/s (the limited frequency range in which many mechanobiological studies have been based), they exhibit vastly distinct viscous behavior at the higher frequency regimes. These wideband viscoelastic spectra may lead to further insights on the multifaceted mechanical transformation in breast cancer and provide previously inaccessible sources of contrast for disease prognostication.

### Revealing wideband frequency–dependent viscoelastic behaviors of bone

**Fig. 4.**
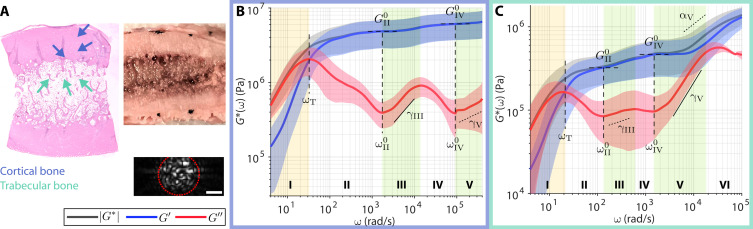
Wideband viscoelastic spectra of compact and trabecular bones. (**A**) Gross photo, H&E slide, and example speckle frame (red dotted line indicates analysis ROI; scale bar, 20 μm) of bovine rib bone cross section. (**B** and **C**) Frequency-dependent |G*(ω)| (black), *G*′(ω) (blue), and *G*″(ω) (red) of cortical and trabecular bones indicated by colored arrows in (A), respectively. Solid curve and shaded area represent means ± SD of *N* = 3 independent measurement at locations indicated by colored arrows on H&E. Arrows and plot outlines are color-coded for cortical (blue) and trabecular (green) bones. (For α and γ, dotted and solid lines indicate that power scaling exponents are <0.5 and ≥0.5, respectively. Values of all annotated spectroscopic parameters are tabulated in table S1.)

Although bone mineral density (BMD) has long served as a diagnostic marker for bone diseases, it is now accepted that BMD is an incomplete index of bone fragility and does not adequately predict fracture risk across disease states (*19*). Furthermore, there is a growing appreciation for the role of structural water on the mechanical integrity of bone in aging and diseases (*75*, *76*). However, mechanical properties of bone are still typically given by elastic moduli, which exhibit little frequency dependence at the macroscale (*13*). Microrheology of bone remains largely unexplored in orthopedic conditions. Leveraging the large dynamic range of WB-SHEAR, not only in the frequency but also in the measurable viscoelastic modulus, we reveal previously unobserved frequency-dependent elastic and viscous behaviors of bone across a wide range of frequency (Fig. 4). (Our low sub-nanometer MSD noise floor enables measurement of extremely small Brownian displacements in high-moduli samples; fig. S3.) Bovine rib bone was cross-sectioned (Fig. 4A) to obtain wideband viscoelastic spectra of the dense cortical bone in the outer shell (Fig. 4B) and the “spongy” trabecular bone in the center (Fig. 4C). Values of all spectroscopic parameters annotated in Fig. 4 are summarized in table S1.

At the microscale, both cortical and trabecular bones exhibit frequency-dependent elastic and viscous behaviors not unlike those of soft tissues. The cortical bone, which is composed of densely packed osteons with aligned fibrous collagen matrix, displays an undulation pattern in the viscous behavior that is characteristic of the fibrous stroma of breast tumors (Fig. 4B). Compared to the fibrous tumor stroma, the undulation of *G*″ begins (i.e., the first local minimum) at a much higher frequency of ω^0^_II_ = 1720 ± 70 rad/s compared to ω^0^_II_ ~ 100 rad/s in (Fig. 3D, G, and I). In addition, although *G*″(ω) follows a similar power scaling behavior (γ_III_ = 0.56 ± 0.09, γ_V_ = 0.2 ± 0.2), |G*(ω)| remains roughly constant over the measured frequency range (i.e., α → 0) with elevated elastic plateau moduli of *G*^0^_II_ = 5 ± 2 and *G*^0^_IV_ = 6 ± 2 MPa. Evidently, the wideband viscoelastic behavior of the cortical bone is more predominantly elastic than that of the fibrous tumor stroma, although both are highly fibrous tissues with interspersed cells (tumors and osteocytes), likely owing to the calcification and mineral contents in the bone. Meanwhile, the trabecular bone, which is composed of less densely arranged trabeculae surrounding soft tissue components such as marrow and blood vessels, exhibit lower elastic plateau moduli (*G*^0^_II_ = 0.3 ± 0.1, *G*^0^_IV_ = 0.5 ± 0.2 MPa) compared to the cortical bone (Fig. 4C). Furthermore, the frequency-dependent elastic and viscous behaviors also bare more resemblance to those of other fibrous soft tissues and scaffolds, with both fluid-to-solid transition and elastic plateau occurring at the same frequency ranges of ω_T_ ~ 20 rad/s and ω^0^_II_ ~ 100 rad/s, respectively. Moreover, the power scaling behaviors of both *G*″(ω) (γ_III_ = 0.1 ± 0.1, γ_V_ = 0.78 ± 0.06) and |G*(ω)| (α_V_ = 0.44 ± 0.07) are notably similar to those of the benign fibrous breast tissue in Fig. 3B.

Although microrheology has traditionally been reserved for the study of complex fluids and soft materials, our results show that bone also exhibits rich frequency-dependent viscoelastic behavior when probed at the microscale. Compared to the relatively constant gigapascal elastic modulus probed at the macroscale (*13*), our results may speak to the substantial contribution of the interstitial fluid components in the porous bone matrix to the viscoelastic behavior within the bone microenvironment. With increasing attention to not only solid components (e.g., collagen and minerals) but also interstitial water in bone (*75*, *76*), examination of both elastic and viscous behaviors of the bone across a wide frequency band is likely to offer unique insights on the microstructural mechanisms underlying orthopedic conditions.

### Mapping spatially variant, wideband viscoelastic spectra in human tumor specimens

**Fig. 5.**
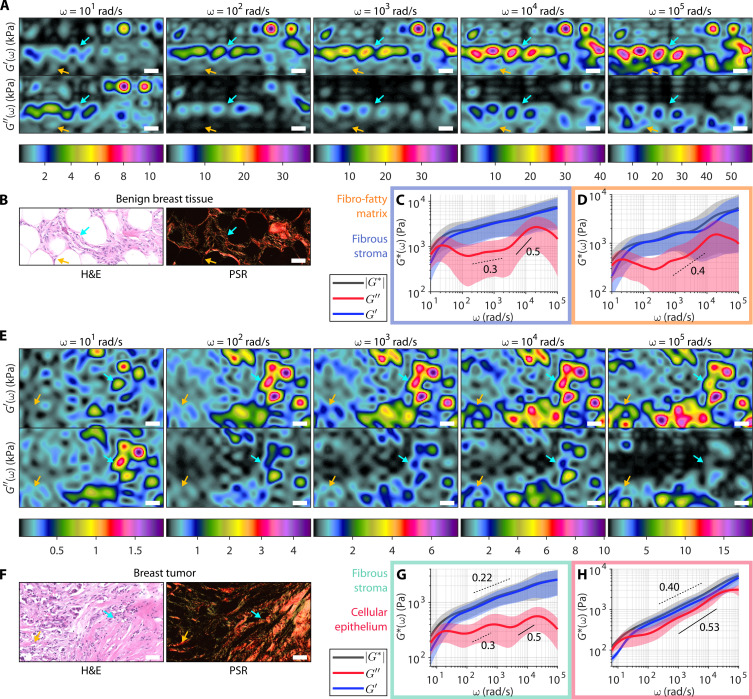
Micromechanical mapping of the wideband viscoelastic spectra in the breast tumor microenvironment. Spatial maps of *G*′(ω) (top) and *G*″(ω) (bottom) in kPa at distinct ω frequencies in (**A**) benign breast tissue and (**E**) breast tumor specimens. Full spectral movies of the frequency-dependent evolution of the maps are provided in movies S1 and S2. (**B** and **F**) Corresponding H&E and PSR slides. Scale bar, 50 μm for all images. Frequency-dependent |G*(ω)| (black), *G*′(ω) (blue), and *G*″(ω) (red) spectra are shown for regions of (**C**) benign fibrous stroma, (**D**) benign fibro-fatty matrix, (**G**) collagenous tumor stroma, and (**H**) cellular tumor epithelium. (See Materials and Methods for histological image analysis to identify these regions.) Solid curve and shaded area represent median ± median absolute difference of *N* = 45, 43, 46, and 5 independent measurement locations for (C), (D), (G), and (H), respectively. (Annotation indicates representative power scaling laws. Full sets of spectroscopic parameters are tabulated in table S5.)

Our results in fibrin constructs, whole blood clots, breast tissues, and bones thus far reveal several characteristic viscoelastic spectral signatures corresponding to different microenvironmental compositions. For instance, the fibrous stroma at the tumor-stroma interface exhibits a unique undulation pattern in *G*″, while the cellular tumor epithelium exhibits greater viscous contribution. We further investigate the presence of these characteristic behaviors in heterogeneous tissue by spatially mapping the wideband viscoelastic spectra in benign breast tissue (Fig. 5A) and breast tumor specimens (Fig. 5E) obtained from patients (MGH IRB no. 2011P000301). To obtain a 2D map, we performed the WB-SHEAR measurement as described and translated the sample laterally at a step size of 25 μm, providing approximately 17 to 39% area overlap between the analysis ROI of two adjacent measurement locations.

In both specimens, the maps of *G*′(ω) and *G*″(ω) reveal microscale spatial variations that are distinct between the elastic and viscous behaviors, as well as at different ω frequencies across the wide frequency band. Notably, measurements spanning 5 decades in ω reveal distinct features that are otherwise not observed in the lower frequency regimes (yellow arrows in Fig. 5, A and E). Corroborating with histological information from hematoxylin and eosin (H&E) and picrosirius red (PSR) staining (see Materials and Methods for histological image analysis), we extracted the representative wideband viscoelastic spectra of the fibrous stroma (Fig. 5C) and fibro-adipose matrix (Fig. 5D) in benign breast tissue, as well as the collagen-rich fibrous tumor stroma (Fig. 5G) and cellular tumor epithelium (Fig. 5H) in breast tumor. Full hyperspectral movies of the frequency-dependent evolution of *G*′(ω) and *G*″(ω) maps in Fig. 5 (A and E) are provided in movies S1 and S2. Values of all spectroscopic parameters discussed below are summarized in table S5.

The benign breast tissue section primarily consists of fibro-fatty matrix (Fig. 5B). The stripe of elevated *G*′ in the WB-SHEAR maps corresponds to the region of fibrous stroma surrounded by adipose (cyan arrow in Fig. 5, A and B). Taking the median of the WB-SHEAR measurements specifically from the region of the fibrous stroma, the wideband viscoelastic spectra exhibit two distinct regimes of *G*″ power scaling, with larger power scaling exponent at higher frequency (0.5 ± 0.2 > 0.3 ± 0.1, n.s.) (Fig. 5C). This behavior is consistent with that of the benign fibrous breast tissue previously observed in Fig. 3B. Compared to the fibrous stroma, the surrounding fibro-fatty matrix exhibits roughly a single *G*″ power scaling law, as well as a lower elastic plateau modulus (1.1 ± 0.6 < 2.2 ± 1.5 Pa; *P* < 0.01) that occurs at a higher frequency (300 ± 100 > 170 ± 80 rad/s; *P* < 0.01) (Fig. 5D). The later onset of the elastic plateau followed by a single *G*″ power scaling law is consistent with the behavior of adipose tissue previously observed in Fig. 3E. We hypothesize that the heterogeneous mix of fibro-adipose here results in a combination of both benign fibrous stromal signature and adipose signature in Fig. 5D.

The breast tumor section consists of collagen-rich fibrous stroma on the right side and a more cellular region on the left side (Fig. 5F). Correspondingly, the WB-SHEAR maps show overall higher moduli on the left side, with the sharp gradient on the *G*′ maps coinciding with the tumor-stroma interface (cyan arrow in Fig. 5, E and F). Taking the median of the WB-SHEAR measurements specifically from the region of the fibrous collagen matrix, the wideband viscoelastic spectra notably exhibit the characteristic undulation pattern in *G*″ (Fig. 5G), consistent with the fibrous tumor stroma previously observed in Fig. 3 (D, G, and I). Similar to Fig. 3D, higher elastic plateau modulus (1.3 ± 0.7 > 0.7 ± 0.3 kPa; *P* < 0.01) and *G*″ power scaling law (0.5 ± 0.1 > 0.3 ± 0.1; *P* < 0.0001) are observed at the higher frequency regime. Compared to the fibrous stroma, the cellular region exhibits a distinctly more viscous behavior, with higher α throughout the higher frequency regimes (0.40 ± 0.04 *>* 0.22 ± 0.06; *P* < 0.01) (Fig. 5H). As expected, the elevated viscous contribution is consistent with that of the cellular tumor epithelium previously observed in Fig. 3 (H and I).

The WB-SHEAR maps of *G*′(ω) and *G*″(ω) at different ω values spanning 5 decades of frequency bring to light the critical need to investigate not only a single measurement of elasticity but also the comprehensive frequency-dependent elastic and viscous behaviors over a wide frequency band. Our results show that distinct histological composites within the tissue specimens are delineated both spatially on the *G*′(ω) and *G*″(ω) maps at different frequency and spectrally by the wideband viscoelastic spectra at different spatial locations. For instance, higher mechanical contrast is observed at ω ≥ 10^4^ rad/s in the benign breast tissue section, including features in the fibro-fatty region at the bottom (yellow arrows) that are only perceptible at ω = 10^5^ rad/s (Fig. 5A). Meanwhile, in the breast tissue section, mechanical contrast at the tumor-stroma interface is clearly defined throughout ω = 10^3^ to 10^5^ rad/s on the *G*′(ω) maps but vary greatly with frequency on the *G*″(ω) maps (Fig. 5E), owing to distinct frequency-dependent viscous behavior of the fibrous stroma versus cellular epithelium (Fig. 5, G and H). By performing 2D spatial mapping, WB-SHEAR not only enables comprehensive analysis of distinct frequency-dependent elastic and viscous behaviors in tissue but also permits 3D hyperspectral (space and frequency) micromechanical imaging at high spatial resolution over an unprecedented wide band of frequencies in heterogeneous tissues.

## DISCUSSION

In this work, we harness the interactions of coherent laser light and thermally driven micromechanical fluctuations of native scattering structures in the biomaterial constituents to obtain “viscoelastic fingerprints” in tissues, biofluids, and biomimetic scaffolds. We demonstrate the capability to access unique spectral signatures of distinct elastic and viscous behaviors over an extended wide frequency band (sub-hertz to sub-megahertz) in a variety of clinically relevant biological specimens. Using lateral scanning, we further demonstrate the unprecedented capability for 2D spatial mapping of these wideband viscoelastic spectra in heterogeneous human tumor specimens. From aqueous solution of fibrinogen to hard bone, our results also demonstrate the capability to measure a remarkable range of shear moduli (~0.1 Pa to over MPa). This large dynamic range is enabled by two main factors unique to WB-SHEAR. First, the high-speed speckle acquisition allows for extremely fast Brownian dynamics in highly compliant samples to be captured, enabling accurate measurements even in low-viscosity solutions. Second, WB-SHEAR inherently exploits the motions of multiple intrinsic tissue structures (as opposed to a single scatterer) to cumulatively produce the detected speckle intensity fluctuation, making it sensitive to extremely small Brownian displacements (see frequency-dependent MSD noise floor in fig. S3) in high-moduli samples (*47*, *53*). In addition, spatial sampling of the speckles, number of individual speckles and temporal frames in the ensemble-averaged *g*_2_(*t*), camera sensor bit depth, and optimal usage of the full camera sensor dynamic range all contribute to the displacement sensitivity and, thus, the maximum measurable shear moduli in each sample. The wideband viscoelastic spectra obtained with WB-SHEAR in biospecimens spanning a vast range of mechanical moduli have the potential to support the study of multiscale mechanics in biological systems, biophysical studies of microstructural dynamics in cells, and ECM, and provide new means to investigate the mechanobiology of various disease pathologies.

From the rheological perspective, WB-SHEAR over a broad range of frequency scales inherently interrogates a comparably wide range of length scales within the structural hierarchy of the material. Cells and extracellular networks that form the tissue at the mesoscale are composed of building blocks at length scales of micrometers down to nanometers and beyond, where structures of smaller characteristic length scales are generally associated with faster dynamical behaviors (*77*–*79*). Thus, viscoelastic moduli that span the lower- to higher-frequency regimes reflect the mechanical behavior of the whole-tissue structure (collective network dynamics) down to the individual constituents (single-filament dynamics) that compose the tissue (*36*, *57*, *58*), despite the fixed spatial resolution of the optical system. At lower frequencies (0.6 < ω < 60 rad/s), our previous studies have demonstrated agreement between G*(ω) measured with SHEAR approach and conventional macrorheology in aqueous solution and biofluids (*44*, *45*), homogeneous hydrogels (*47*), and breast tissues (*53*). Here, WB-SHEAR in fibrin constructs (Fig. 1D) shows that G*(ω) most closely corresponds to macrorheology measurement at the elastic plateau (fig. S2), in agreement with the interpretation that the elastic plateau is governed by collective network elasticity (*36*, *57*, *58*). At the elastic plateau, we find that *G*″ is approximately 10% of *G*′ (Fig. 1D), which is also consistent with existing literature from macrorheology in various types of tissues and reconstituted ECMs (*23*). Beyond the elastic plateau, WB-SHEAR provides additional rheological information that is inaccessible to conventional macrorheology.

From the biophysical perspective, WB-SHEAR reveals frequency-dependent behaviors that may facilitate future studies of physics-based biomaterial models via constitutive modeling. Despite vastly diverse spectral signatures across different types of tissues, we show that the wideband viscoelastic spectra can be empirically divided into smaller frequency regimes, within which the frequency-dependent behavior follows aspects of characteristic behaviors that have been previously observed in biological samples and predicted by biophysical models. Taking the fibrin construct (Fig. 1D) as an example, the transition from the low-frequency viscosity-dominant behavior (regime I) to the elasticity-dominant behavior that results in an elastic plateau at the intermediate frequencies (regime II) has been observed in partially cross-linked hydrogels (*39*, *62*) and may likely be reminiscent of the behavior of a Maxwell body (*80*, *81*). Meanwhile, the high-frequency power scaling behaviors of *G*″ and |G*| (regimes IIIa and IIIb) have been observed via microrheological measurements in homogeneous hydrogels (*39*, *62*, *63*, *65*) as well as cytoskeletal and extracellular networks of various cells (*40*, *64*, *66*). Here, our results further show that these regime-wise behaviors tend to repeat beyond the initial regimes I to III in complex tissue specimens. In this study, the empirical division of the wideband spectra into smaller frequency regimes allows the otherwise complex spectral behaviors to be described by a set of quantitative metrics (i.e., the spectroscopic parameters). Beyond this study, a combination of correlative analysis between WB-SHEAR and microstructural metrics, as well as constitutive modeling of the wideband viscoelastic spectra, may provide a way to experimentally elucidate the biophysical origins of the emergent multiscale viscoelastic behaviors of complex ECM and tissues through future studies.

From the mechanobiological perspective, WB-SHEAR provides unique viscoelastic fingerprints that characterize a wide array of tissue microenvironments. Several types of characteristic spectral signatures emerge from our analysis of fibrin constructs, whole-blood clots, breast tissues, and bones. The first type is observed in acellular fibrin construct (Fig. 1D) and benign fibrous breast tissue (Figs. 3B and 5C), where the power scaling of *G*″∝ω^γ^ in regime III follows two distinct scaling laws, with a steeper increase (i.e., larger γ) at higher frequency. This type of behavior bears most resemblance to previous literature in cell-free fibrous scaffolds (*31*, *39*). In contrast, the second type is observed in highly cellular tissues, including the tumor epithelium (Figs. 3, H and J, and 5H) and whole-blood clots packed with RBCs and platelets (Fig. 2). These spectra are characterized by a relatively more viscous behavior, where *G*″ remains close to, or even exceeds, *G*′ at higher frequencies. Given the predominantly cellular microenvironments, we hypothesize that this type of behavior is dominated by the thermal dynamics of cell surfaces, which are surrounded on either side by the cytoplasm and the interstitial fluid. The third type is characteristic to adipose tissue (Fig. 3E), where the delayed elastic plateau and the single steep power scaling of *G*″ result in a distinct inverted triangle shape of the spectra. These spectra may reflect the viscoelastic behavior of lipid droplets in the intracellular space of densely packed adipocytes. The fourth type is observed where fibrous stroma meets cells, including the breast tumor–stroma interface (Figs. 3, D, G, and I, and 5G) and the cortical bone (Fig. 4B). These spectra exhibit a characteristic *G*″ undulation, where multiple minima are repeatedly reached after ~1 decade of power scaling behavior. We hypothesize that the multiple minima may be attributed to distinct thermal dynamics of multiple interacting constituents of cells and surrounding fibrillar network, given that this behavior is, to the extent of our results, strictly observed where cells interface with the fibrous ECM.

The ability to comprehensively investigate wideband viscoelastic spectra of biospecimens that span a vast range of mechanical modulus values is by itself invaluable in mechanobiology and related fields. Nevertheless, the importance of investigating the spatial variation in the micromechanical properties of heterogeneous tissue and ECM cannot remain understated. Using 2D lateral scanning, we show that WB-SHEAR also enable 3D hyperspectral micromechanical imaging of spatially variant, wideband viscoelastic spectra at high spatial resolution (scanning step size of 25 μm in this work) in microscopically heterogeneous tissue. The WB-SHEAR maps of *G*′(ω) and *G*″(ω) at distinct ω frequencies reveal micromechanical heterogeneities within the breast microenvironment that are well-corroborated by the histological compositions via H&E and PSR staining (Fig. 5). Contrasting features observed on the maps of *G*″ differ from those of *G*′, highlighting the importance of distinguishing the contributions of elastic and viscous behaviors to the overall “stiffness” of biological tissues. Moreover, structures such as the tumor-stroma interface in Fig. 5E are most well delineated at the kilohertz to megahertz frequency range, whereas certain features of viscosity contrast are only perceptible at tens of hertz range, reinforcing the idea that cells sense different mechanical cues at distinct “probing” frequencies. The sharp elasticity contrast along the tumor-stroma interface at high frequency and the distinct viscosity contrast in the stroma at low frequency (Fig. 5E) may be related to the simultaneous high-frequency stiffening and low-frequency fluidization in the in vitro model of cancer cell–seeded collagen matrices (*31*). Thus, spatial mapping with WB-SHEAR offers a unique capability to quantitatively investigate the micromechanical heterogeneity in both the elastic and viscous moduli, as well as their wideband frequency–dependent behaviors, in fresh unprocessed tissue specimens.

The active role of living cells in mediating the mechanical properties of the ECM is an important subject of investigation for mechanobiological studies. Cellular activity can bring the microenvironment under study out of thermal equilibrium, precluding accurate reconstruction of G*(ω) from the measured MSD via the GSER. Previous live-cell imaging studies in 3D cell culture systems (*31*, *82*) as well as fresh tissue specimens (*83*), however, show that perceptible dynamics associated with cellular activities tend to occupy the lower-frequency range. In particular, Krajina *et al.* (*31*) found that thermal (Brownian) dynamics remains the dominant contribution to the measured MSD at ω ~ 10 rad/s and beyond in the presence of living human fibroblasts and breast cancer cell lines. In this context, the wide frequency band up to sub-megahertz supported by WB-SHEAR offers a critical advantage in enabling passive microrheological measurements in the presence of living cells over several more decades at the higher-frequency regimes. In practice, the frequencies at which active cellular dynamics brings WB-SHEAR out of thermal equilibrium can be readily identified from the measurement of α(ω), where α > 1 indicates the presence of actively driven motions (*31*, *55*). This capability presents an opportunity to apply WB-SHEAR to comprehensively investigate the cell-mediated spatiotemporal mechanical remodeling of the ECM, not only in in vitro cell culture systems similar to (*84*–*87*) but also in whole-tissue specimens.

There are some notable limitations in the present study. First, we have estimated a single-particle size for each type of sample based on polarization-sensitive speckle attributes (*46*) and literature values of the sizes of various scattering structures (see Materials and Methods and table S3). This could lead to variability in the computed moduli values (Eq. 3) in heterogeneous tissue specimens with spatially varying scattering particle size. On the basis of our previous study, we anticipate a variability of up to 35% in the scattering particle radii across heterogeneous benign and cancerous breast tissue specimens (*53*). Nevertheless, as shown in both our previous study (*53*) and Fig. 5 here, spatially variant micromechanical signatures can be appreciated even when assuming a uniform scattering particle size. Our polarization-sensitive particle size estimation (*46*, *53*) can be implemented at each measurement location and, furthermore, at each pixel to substantially minimize the variance in the computed moduli due to spatially varying particle size. Second, WB-SHEAR provides a depth-integrated measurement from all scattering tissue structures within the volume interrogated by the multiply-scattered light; it does not currently support depth-resolved imaging capability. However, depth information may be extracted from the speckle time series using inverse problem approaches to analyze speckle decorrelation as a function of radial distance from the illumination center (*88*). Alternatively, in samples where spatial variation as a function of depth is of interest, additional procedures such as reorienting and/or slicing the specimen should be considered. Third, WB-SHEAR in its current implementation is inefficient for high-resolution spatial mapping. With focused illumination, WB-SHEAR maps were constructed via point scanning (by translating the sample), which limited the spatial resolution to the scanning step size (25 μm in this study). Furthermore, we computed the ensemble-averaged intensity autocorrelation function over an area of 36 to 75 μm in diameter around each illumination “point.” Thus, the spatial resolution of the reconstructed G* map also bears contribution from the spatial averaging in addition to the primary limiting factor of the scanning step size. An alternative implementation would be with wide-field illumination and a pixel-wise reconstruction via a sliding Gaussian spatial averaging window. This approach has been used in our previous SHEAR studies, demonstrating high-resolution mapping of |G*| in microfabricated phantoms (*47*) and breast tissue specimens (*53*) using an averaging window width of 9 μm. In principle, the smallest possible spatial averaging window is the size of a single speckle grain (i.e., the optical diffraction limit). However, the reduced spatial averaging must be compensated for by acquiring a longer speckle time series to maintain statistical accuracy.

The unprecedented ability to conduct comprehensive “viscoelastic fingerprinting” as well as 3D hyperspectral micromechanical imaging in biospecimens spanning a broad range of mechanical moduli presents ample opportunities for mechanobiological discoveries in a number of pathologies. One exciting opportunity is in the fundamental investigation of cancer mechanopathology. Recent findings from in vitro cell culture studies suggest that cells induce mechanical transformations in the ECM that are both spatially variant (*84*, *87*) and frequency dependent (*31*). By enabling comprehensive investigation into spatially variant, frequency-dependent viscoelastic behavior in real tissue of clinical tumor specimens, WB-SHEAR can help advance our understanding of such phenomena as well as how they relate to malignancy in patients. Toward clinical translation, the spectroscopic parameters extracted from the wideband viscoelastic spectra can be used to investigate the association of tumor mechanical indices with immunohistochemical biomarkers, clinical prognostic indicators, and treatment outcome in patients with cancer. In this context, WB-SHEAR can greatly enhance clinical studies such as in (*53*, *74*) by providing a substantially more comprehensive set of mechanical indices derived from both spatial and spectral signatures in the wideband viscoelastic spectra. Another exciting opportunity is in the investigation of degenerative mechanical transformations in bone, particularly the viscous behavior and role of bone structural water (*75*, *76*), associated with orthopedic diseases. Toward clinical translation, WB-SHEAR can provide comprehensive biomechanical evaluation for the development of novel tissue engineering solutions for regenerative purposes. The broad applicability of WB-SHEAR opens the door for microrheological investigation in previously unexplored disease systems such as bone.

## MATERIALS AND METHODS

### WB-SHEAR system and data acquisition

Schematics of the optical setup for the WB-SHEAR system are shown in Fig. 1A. A fiber-coupled diode laser with wavelength 637 nm (Coherent, OBIS FP 637LX) was collimated, linearly polarized, resized to a beam diameter of 1 mm, and directed into the sample by a nonpolarizing beam splitter (the remaining beam collected by a beam trap). The illumination beam was focused by an objective lens (convex doublet, focal length 30 mm) to a spot size of 14 μm at the sample. Backscattered light was collected by the same objective lens through an open aperture of 9 mm in diameter and then captured by a high-speed CMOS camera (Photron, Mini AX200 type 900 k) through another linear polarizer and a tube lens (focal length, 400 mm). Acquisition of speckle time series was accomplished with a manufacturer-provided camera control software (Photron FASTCAM Viewer 4), which enabled control of camera ROI, frame rate, and exposure time.

Compared to the systems described in our previous work (*44*–*47*), notable advances were implemented here to enable WB-SHEAR. The absolute upper- and lower-frequency limits of the measured viscoelastic spectra are determined by the acquisition frame rate, *F*_s_, and duration, τ, of the speckle time series according to 1/τ < ω < *F*_s_. Thus, extending the measurement up to the sub-megahertz frequency range necessitates a high-speed camera that can capture speckle images at the sub-megahertz frame rate. The Photron Mini AX200 camera can support up to 540,000 frames/s (with a sensor bit depth of 12 bits and ROI of 128 × 32 pixels), several orders of magnitude faster than typical CMOS cameras for microscopy applications. However, the Mini AX200 camera also comes with a much larger sensor pixel size of 20 μm × 20 μm, which presents a caveat for achieving sufficient spatial sampling to capture fully developed speckles without compromising photon collection (e.g., by closing the aperture on a zoom lens). Using a combination of illumination beam size, collection aperture size, and focal lengths of objective and tube lenses, we designed the optical system to achieve a pixel-to-speckle size ratio of 3.5 pixels/speckles (along one dimension). We found this spatial sampling optimal for ensuring both robust imaging of fully developed speckles and maximizing the number of individual speckles within the analysis ROI for ensemble averaging (see reconstruction methods in the following subsection). Speckle frames were acquired with a sensor dynamic range of 12 bits. Furthermore, the high-frame rate acquisition affords shorter exposure time, necessitating higher illumination power to capture the speckle intensity fluctuation with sufficient signal-to-noise ratio. The Coherent OBIS FP 637LX laser diode provides a full power of 48 mW at the sample. We used the full power except for measurements in whole blood (Fig. 2), where a neutral density filter was placed in the illumination arm to achieve 10 mW at the sample to avoid excessive absorption by hemoglobin.

WB-SHEAR data were acquired according to the following procedure. Samples were positioned with the focal plane of the objective lens just below the sample surface. For each measurement in Figs. 1 to 4, we identified the lowest possible camera exposure time at which the captured speckle intensity still occupies the full available dynamic range of the camera sensor. At the maximum allowable frame rate for a given exposure time, speckle time series was acquired over a sensor ROI of 128 × 32 pixels for a duration of 5 s. For the spatial maps in Fig. 5, the samples we placed on a two-axis motorized stage (Newport, VP-25XA) and laterally translated in a 2D grid pattern at incremental steps of 25 μm in both directions, spanning a scan area of 0.5 mm by 0.2 mm (Fig. 5A) and 0.5 mm by 0.275 mm (Fig. 5E). At each lateral location, speckle time series was acquired at a frame rate of 240 kHz (Fig. 5A) or 120 kHz (Fig. 5E) over a sensor ROI of 128 × 64 pixels for at least 40,000 frames. The camera exposure time was adjusted at each lateral location to avoid any sensor saturation.

### Reconstruction of frequency-dependent G*(ω)

All computation was implemented in MATLAB 2022a. First, ensemble-averaged intensity autocorrelation function, *g*_2_(*t*), was computed from the acquired speckle time series using a contrast-normalized approach described in (*89*)

$$g_{2}\left(t\right)=\frac{g_{2,\text{raw}}\left(t\right)-1}{g_{2,\text{raw}}\left(t=0\right)-1}+1;g_{2,\text{raw}}\left(t\right)=\frac{\langle I\left(t+t_{0}\right)I\left(t_{0}\right)\rangle }{\langle I\left(t_{0}\right)\rangle \langle I\left(t+t_{0}\right)\rangle }$$ (1)

where *I* and *t* denote speckle intensity and autocorrelation time, respectively. ⟨⟩ denotes an ensemble average in space and time. Here, the ensemble includes all spatial pixels in a circular analysis ROI concentric to the illumination center and extending 1/*e* radius of the DRP (obtained by temporally averaging all frames in the speckle time series) and all pairs of temporal frames separated by time *t*. For the samples presented here, the analysis ROIs (indicated by red dotted circles in Figs. 2 to 4) have diameters in the range of 36 to 75 μm. This ensures optimal speckle contrast in the ensemble while maximizing the number of individual speckles and temporal averaging.

Then, time-dependent MSD, ⟨Δ*r*^2^(*t*)⟩, was obtained via an empirical approximation of the DWS formulation (*44*, *45*)

$$g_{2}\left(t\right)-1=\text{exp}\{-2\gamma {\left[k^{2}\langle \Delta r^{2}\left(t\right)\rangle \right]}^{\zeta }\}$$ (2)

where *k* denotes wave number in the medium, while γ and ζ are experimental constants that account for the optical properties of the sample. The constants γ and ζ were obtained from a lookup table derived via Monte Carlo ray tracing for a given sample optical properties, which were estimated by fitting the experimentally measured radial DRP (obtained by temporally averaging the speckle time series) in each sample to the diffusion theory (fig. S1), as previously described (*44*, *45*). For fibrin construct (Fig. 1), whole-blood clots (Fig. 2), and bone (Fig. 4), DRP was measured at three locations to provide average γ and ζ values for each sample (for bone, three locations each in the cortical and trabecular regions). For breast tissues (Figs. 3 and 5), the speckle time series from each measurement location was directly temporally averaged to obtain the DRP, providing γ and ζ values for each location. This approach allows the SHEAR approach to navigate a range of unknown intrinsic optical properties in scattering biological tissues, unlike conventional DLS and DWS approaches, which are limited to either the single-scattering or the multiple-scattering extremes, respectively (*44*, *45*). Notably, a combination of γ = 2/3 and ζ = 1 reduces Eq. 2 to that of the DLS formulation, while γ = 5/3 and ζ = 0.5 are consistent with the DWS formulation (at 180° backscattered configuration). In the present study, the values of γ and ζ fall between the two limits based on the optical properties of the samples summarized in table S3.

In principle, G*(ω) can be obtained directly from the temporal Fourier transformation of the MSD via the GSER. In practice, since MSD is measured over a finite time domain, we used an algebraic approximation of the GSER (*54*–*56*):

$$\begin{array}{c} G^{*}\left(\omega \right)=\frac{k_{B}T}{\pi a\Gamma [1+\alpha \left(\omega \right)[\langle \Delta r^{2}\left(\omega \right)\rangle }\text{exp}[i\frac{\pi \alpha \left(\omega \right)}{2}];\\ \alpha \left(\omega \right)={\frac{\partial \text{log}\langle \Delta r^{2}\left(t\right)\rangle }{\partial \text{log}\left(t\right)}|}_{t=1/\omega } \end{array}$$ (3)

where *k*_B_, *T*, and *a* denote the Boltzmann constant, temperature, and scattering particle radius, respectively. We note that in tissues, light scattering structures (e.g., cell nuclei, organelles, cell membranes, and various ECM proteins) can span a range of underlying microstructural length scales with varying number density and scattering cross section. Thus, *a* in Eq. 3 represents the average sphere-equivalent radius of the structures that most dominantly contribute to the captured speckle images. For samples with unknown scattering particle size, *a* can be experimentally estimated from a combination of the azimuthal DRP and the relative speckle decorrelation rate of parallel versus perpendicularly polarized component of the backscattered light, as previously described (*46*, *53*). Notably, *a* merely provides a scaling factor to the magnitude of G*(ω) and has no effect on its frequency-dependent variation or the relative contribution of elastic and viscous components. The average scattering particle radii used in the present results are based on literature values of the radii of scattering structures, such as RBC radius blood and mineral crystal size in bone, and experimentally estimated from polarized speckle patterns for breast tissue and fibrin as summarized in table S3.

This approximation describes the MSD with a frequency-dependent power scaling law, α(ω), where ⟨Δ*r*^2^(*t*)⟩∝*t*^α(ω)^ at *t* = 1/ω. For optimal sampling in the frequency domain and noise performance in the log-log derivative, Eq. 3 was executed by first resampling the MSD linearly in the log-space ω domain with 30 points per decade to obtain ⟨Δ*r*^2^(ω)⟩ and then computing the linear regression of log⟨Δ*r*^2^(*t*)⟩ with respect to log(*t*) over a rolling temporal window of width 7 points centered at *t* = 1/ω to obtain α(ω). Then, α(ω) was smoothed by a moving-average filter with a window size of 15 points before G*(ω) was obtained via Eq. 3. Last, G*(ω) was further smoothed by another moving-average filter of the same window size to produce the final wideband viscoelastic spectra shown in Figs. 1 to 5. For the 2D spatial mapping, the final *G*′(ω) and *G*″(ω) values at different ω from each of the lateral measurement locations were compiled into 2D maps. For smooth visualization, each map was spatially upsampled by a factor of 7 via 2D Fourier transformation and zero-padding for the final displays in Fig. 5 (A and E) and movies S1 and S2.

### Extraction of spectroscopic parameters from G*(ω)

All computation was implemented in MATLAB 2022a. Spectroscopic parameters were extracted from the final |G*(ω)|, *G*′(ω), *G*″(ω), and α(ω) spectra reconstructed for each WB-SHEAR measurement.

1. Transition frequency, ω_T_ (regimes I and III): value of ω at which α(ω) = 0.5.

2. Plateau frequency, ω^0^ (regimes II, IV, and VI): value of ω at which α(ω) is at its local minima, which also corresponds to local minima of *G*″(ω).

3. Plateau modulus, *G*^0^ (regimes II, IV, and VI): value of *G*′(ω) at ω = ω^0^.

4. Power scaling law of *G*″(ω), γ (regimes III, V, and VII): log-log slope from a linear regression of log[*G*″(ω)] with respect to log(ω) within each regime.

5. G″(ω) power scaling transition frequency, ω_γ_ (regime III): value of ω at the inflection point of the log-log derivative ∂log[*G*″(ω)]/∂log(ω) within regime III.

6. Power scaling law of |G*(ω)|, α (regimes III and V): log-log slope from a linear regression of log(|G*(ω)|) with respect to log(ω) within each regime.

For Figs. 1 to 4, spectroscopic parameters extracted from individual WB-SHEAR measurements in each plot were averaged and means ± SD are reported in tables S1 and S2. For Fig. 5, median of the spectroscopic parameters extracted from individual WB-SHEAR measurements in each plot were computed and median ± median absolute difference are reported in table S5.

### Sample preparation

Fibrin constructs (Fig. 1) were prepared with human fibrinogen plasminogen–depleted (Enzyme Research Laboratories, FIB 1) and human α-thrombin (Enzyme Research Laboratories, HT 1002a) in HBS buffer [20 mM Hepes, 135 mM NaCl, and 5 mM CaCl_2_ (pH 7.4)] at a final concentration of fibrinogen (5 mg/ml) and thrombin (2 U/ml). Fibrinogen and thrombin were separately diluted in HBS buffer to 2× the final concentrations. A volume of 150 μl of each diluted solution was added to a 96-well plate and thoroughly mixed by repeated pipetting. The plate was sealed with parafilm, while the samples were allowed to polymerize at room temperature for 1 hour. Solution of unpolymerized fibrinogen (Fig. 1E) was prepared in HBS buffer at the same final concentration of fibrinogen (5 mg/ml) with no thrombin. Polystyrene microspheres with diameter of 3 μm (Bangs Laboratories, PC05003) were surface-functionalized with polyethylene glycol (Creative PEGWorks, mPEG-Amine, MW 5k) (*90*) and added to the solution to provide scattering particles in the absence of the fibrin network structure.

Whole-blood clots (Fig. 2) were prepared with patient whole-blood samples from the MGH Core Laboratory (MGH IRB no. 2017P000419). The fibrinogen content reported by the Core Laboratory was 5.15 mg/ml for high-FIB and 2.12 mg/ml for low-FIB. Clotting was initiated by adding kaolin (Sigma-Aldrich, K1512) and CaCl_2_ to whole blood at a final concentration of 3 μg/ml kaolin and 14 mM CaCl_2_. The clots were prepared in a 96-well plate with a total volume of 280 μl in each well. The plate was sealed with parafilm, while the samples were allowed to clot at room temperature for 1 hour.

Breast tissues (Figs. 3 and 5) were obtained from the MGH Pathology Unit following surgical tumor resection (MGH IRB no. 2011P000301). The specimens were stored in phosphate buffer saline at 4°C and measured fresh within 24 hours of resection. The specimens were removed from saline, placed on top of saline-soaked gauze in a petri dish, marked with ink at four corners for subsequent coregistration with histology, and allowed to warm to room temperature. The specimens were placed on a two-axis vernier micrometer stage for WB-SHEAR measurement, where the measurement locations (arrows in Fig. 3, A, C, and F) were tracked with respect to the ink marks. Measurements were taken at room temperature. Following the measurement, the specimens were fixed in 10% neutral-buffered formalin, paraffin-embedded, sectioned, and stained with H&E and PSR.

Bone samples (Fig. 4) were obtained from cross sections of bovine rib and stored at 4°C. The specimens were removed from the refrigerator, placed on top of saline-soaked gauze in a petri dish, marked with ink at four locations for subsequent coregistration with histology, and allowed to warm to room temperature. Similar to breast tissues, the WB-SHEAR measurement locations (arrows in Fig. 4A) were tracked with respect to the ink marks via vernier micrometer. Following the measurement, the specimens were fixed in 10% neutral-buffered formalin, decalcified, trimmed around the ink marks, paraffin-embedded, sectioned, and stained with H&E.

### Histological image analysis

Tissue sections stained with H&E were digitized with brightfield microscope (Hamamatsu, NanoZoomer Slide Scanner) using a 40× objective. Tissue sections stained with PSR were digitized with polarized light microscope (Olympus, BX43) using circular cross-polarization and a 20× objective. The digitized H&E and PSR images were used to identify the location of WB-SHEAR measurements that primarily correspond to the fibrous stroma, cellular epithelium, and fibro-fatty matrix from the 2D maps in Fig. 5. H&E images were segmented into binary masks of fibrous tumor stroma (*F*_HE_) and cellular tumor epithelium (*T*_HE_) via color space analysis on the L*a*b* color space (MATLAB 2022a, Color Thresholder). For the benign breast tissue in Fig. 5B, binary mask of adipose (*A*_HE_) was further segmented from the remaining areas (i.e., not belonging to either the fibrous stroma or the tumor epithelium) by excluding small areas corresponding to the interstitial space. PSR images were segmented into binary mask of fibrous collagen matrix (*F*_PSR_) via thresholding of pixel intensity. The segmented *F*_HE_, *T*_HE_, *A*_HE_, and *F*_PSR_ binary masks were further divided into individual square grids with dimension 25 μm by 25 μm, corresponding to the WB-SHEAR measurement locations that form the 2D spatial maps. Then, the fraction of “true” *F*_HE_, *T*_HE_, *A*_HE_, and *F*_PSR_ pixels within a 25 μm–by–25 μm square were calculated for each of the measurement locations. On the basis of these fractions, each WB-SHEAR measurement location was categorized as being primarily fibrous stroma, cellular epithelium, or fibro-fatty matrix according to the criteria outlined in table S4.

### Statistical analysis

Comparisons were made between different spectroscopic parameters extracted from the wideband viscoelastic spectra that form the 2D spatial maps in Fig. 5. Mann-Whitney *U* test was performed using two-sided Wilcoxon rank sum test in MATLAB 2022a to compare the medians of two spectroscopic parameters. Significant difference between medians was decided at the confidence level of 0.05. *P* values are reported in table S5 (exact values) and in the text describing the comparisons (n.s. indicates not significant). The numbers of samples *N* (i.e., number of independent WB-SHEAR measurement locations) are reported in the caption of Fig. 5 and table S5.

## Supplementary Materials

### This PDF file includes:

Figs. S1 to S3 Tables S1 to S5 Legends for movies S1 and S2 References
