# Supplementary material for: Laser speckle rheological microscopy reveals wideband viscoelastic spectra of biological tissues
Source: Sci Adv. 2024 May 8;10(19):eadl1586. doi: 10.1126/sciadv.adl1586 (PMC11078189; doi:10.1126/sciadv.adl1586)
Supplement: Supplementary file 1 — Figs. S1 to S3 Tables S1 to S5 Legends for movies S1 and S2 References [file sciadv.adl1586_sm.pdf]

Science Advances
AAAS

Supplementary Materials for

Laser speckle rheological microscopy reveals wideband viscoelastic spectra of biological tissues

Nichaluk Leartprapun *et al.*

Corresponding author: Seemantini K. Nadkarni, snadkarni@mgh.harvard.edu

Sci. Adv. **10**, eadl1586 (2024)
DOI: 10.1126/sciadv.adl1586

The PDF file includes:

Figs. S1 to S3
Tables S1 to S5
Legends for movies S1 and S2
References

Other Supplementary Material for this manuscript includes the following:

Movies S1 and S2

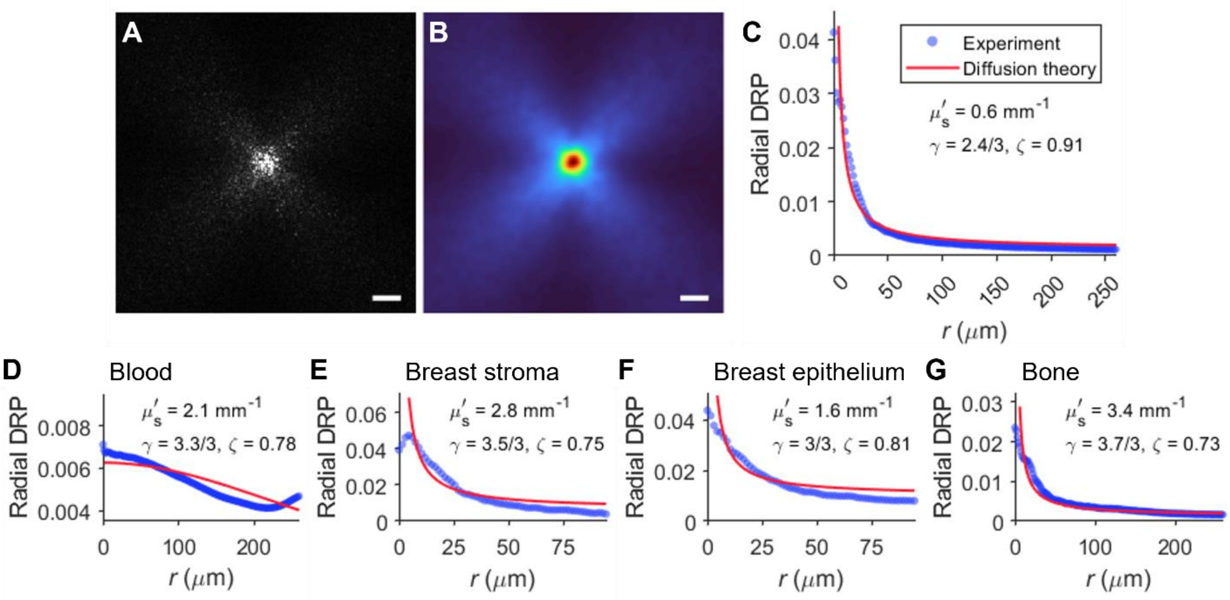

Fig. S1. Estimation and compensation of native optical properties of biological samples. (**A**) and (**B**) An example frame from a speckle time series and the corresponding diffused reflectance profile (DRP) from its temporal average obtained in purified fibrin construct, respectively. Scale bar: 50 μm (**C**) Radial profile (averaged across all azimuth angle) of the DRP of fibrin in (B) and the corresponding curve-fit from photon diffusion theory, providing an estimate of the reduced scattering coefficient, μ_s'. A lookup table generated via Monte Carlo ray tracing provides the values of constants γ and ζ in Eq. 2 for the estimated μ_s' (*44*, *45*). (**D**)–(**G**) An example of μ_s' estimation and the corresponding γ and ζ values in each type of biological specimens presented in this manuscript.

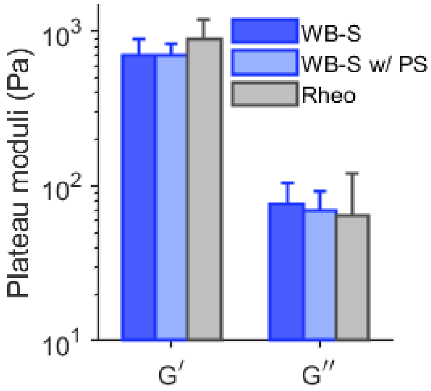

Fig. S2. Agreement with shear rheometry in fibrin construct. Bar plots of *G′* and *G″* of measured by WB-SHEAR at the elastic plateau ($\omega=\omega^0_{\mathrm{II}}$) in pure fibrin construct (dark blue, Fig. 1D) and fibrin construct with embedded polystyrene (PS) microspheres (light blue), in comparison to parallel-plate shear rheometry (TA Instruments, AR-G2 rheometer) at ω=1 rad/s (gray). Bar height and error bar represent mean±standard deviation of N=9 technical (3 independent locations in each of 3 biological replicates) and 5 biological replicates for WB-SHEAR and rheometry, respectively. Rheometry measurement was conducted at room temperature with a 40-mm diameter parallel plate and 0.5% strain. Fibrin precursor solution, prepared as described in Methods, was loaded onto the plate to a gap of 1 mm and allowed to polymerize for at least 30 minutes. There is no statistically significant difference among the three measurements. These results demonstrate the ability of WB-SHEAR to navigate different sample optical properties (i.e., pure fibrin and fibrin with added scattering particles) as well as the agreement with conventional shear rheometry.

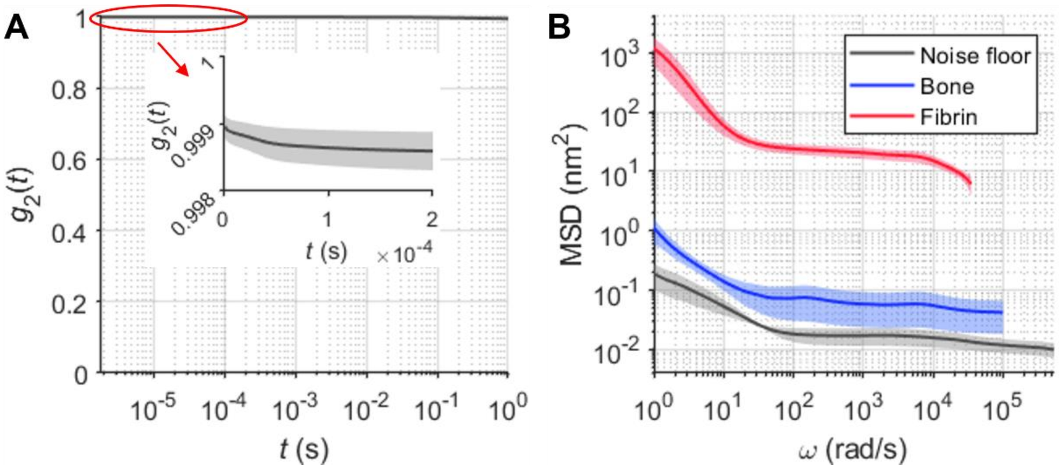

Fig. S3. Characterization of frequency-dependent MSD noise floor. Measurement sensitivity of the WB-SHEAR system in measuring the MSD of scattering particles was characterized by measuring the MSD noise floor in a rigid block of a reflectance standard (Labsphere, USRS-99-010), following the same measurement and reconstruction procedures outlined in Methods. (**A**) Ensemble-averaged $g_2(t)$ and (**B**) frequency-dependent MSD measured in a rigid block (black). Solid curve and shaded outline represent mean±standard deviation of N=5 technical replicates. MSD measured in the fibrin construct (red, Fig. 1) and cortical bone (blue, Fig. 1B) are also provided for comparison. In soft fibrin construct, the MSD in soft fibrin constructs is multiple orders of magnitude larger than the noise floor. In the cortical bone, the highest moduli measured in this study, the MSD is >3 folds above the noise floor throughout the entire measured frequency band. Thus, the MSD measured by WB-SHEAR in bone and other biological specimens in this study is predominantly due to the Brownian excursions of intrinsic scattering particles associated with the viscoelastic behavior of the surrounding microenvironment.

Parameters	Fibrin construct	Whole blood clot		Bone	
		High FIB	Low FIB	Cortical	Trabecular
	(Fig. 1D)	(Fig. 2A)	(Fig. 2B)	(Fig. 4B)	(Fig. 4C)
Regime I					
ω_T	20±3	0.36±0.09	-	30±20	23±7
Regime II					
ω^0_{II}	300±100	3.1±0.7	0.8±0.2	1720±70	140±20
G^0_{II}	0.7±0.2	0.6±0.2 Pa	0.3±0.2 Pa	5000±2000	300±100
Regime III					
γ_{IIIa}	0.4±0.1	0.60±0.01	0.68±0.05	0.56±0.09	0.1±0.1
α_{IIIa}	-	-	-	-	0.16±0.03
$\omega_{T,III}$	-	110±50	40±30	-	-
ω_γ	2420±70	160±30	200±30	-	-
γ_{IIIb}	1.2±0.1	0.83±0.01	0.80±0.02	-	-
α_{IIIb}	0.50±0.09	0.83±0.01	0.80±0.02	-	-
Regime IV					
$\omega^0_{IV}\times10^3$	-	-	-	90±30	1.5±0.2
G^0_{IV}	-	-	-	6000±2000	500±200
Regime V					
γ_V	-	-	-	0.2±0.2	0.78±0.06
α_V	-	-	-	-	0.44±0.07

Table S1. Wideband spectroscopic parameters of fibrin constructs, whole blood clots, and bones. Spectroscopic parameters extracted from frequency-dependent $|G^*(\omega)|$, $G'(\omega)$, $G''(\omega)$, and $\alpha(\omega)$ spectra measured by WB-SHEAR. Unless otherwise specified, values of G and ω are reported in units of kPa and rad/s, respectively. Values indicate mean±standard deviation of N=9 (fibrin construct, 3 independent locations in each of 3 biological replicates), 5 (whole blood clot, 5 independent locations in 1 biological replicate), and 3 (bone, 3 independent locations in each specimen) measurements.

Parameters	Benign	Tumor					
	Fibrous	Adipose	Fibrous stroma			Cellular epithelium	
	(Fig. 3B)	(Fig. 3E)	(Fig. 3D)	(Fig. 3G)	(Fig. 3I)	(Fig. 3J)	(Fig. 3H)
Regime I							
ω_T	13±4	100±30	30±10	18±3	16±2	20±10	8±7
Regime II							
ω^0_{II}	210±20	1500±200	100±20	86±3	100±30	100±40	50±20
G^0_{II}	3.5±0.6	1.27±0.06	4.6±0.9	0.90±0.09	0.61±0.05	0.5±0.1	0.3±0.2
Regime III							
γ_{IIIa}	0.20±0.03	0.8±0.1	0.33±0.09	0.44±0.03	0.4±0.1	0.45±0.02	0.62±0.03
α_{IIIa}	-	0.44±0.05	0.22±0.06	0.27±0.05	0.25±0.05	0.40±0.01	-
$\omega_{T,III}$	-	-	-	-	-	-	-
ω_γ	2000±200	-	-	-	-	-	900±200
γ_{IIIb}	0.73±0.05	-	-	-	-	-	0.51±0.09
α_{IIIb}	0.33±0.05	-	-	-	-	-	0.51±0.09
Regime IV							
$\omega^0_{IV}\times10^3$	200±100	-	2.3±0.7	3.1±0.3	2±1	3.3±0.4	-
G^0_{IV}	13±2	-	8±2	2.1±0.4	1.2±0.6	1.7±0.4	-
Regime V							
γ_V	0.5±0.1	-	0.55±0.06	0.58±0.06	0.3±0.2	0.7±0.1	-
α_V	-	-	0.26±0.01	0.28±0.07	0.26±0.06	0.45±0.07	-
Regime VI							
$\omega^0_{VI}\times10^3$	-	-	130±40	90±30	-	-	-
G^0_{VI}	-	-	18±5	5±2	-	-	-
Regime VII							
γ_{VII}	-	-	0.6±0.3	0.35±0.01	-	-	-

Table S2. Wideband spectroscopic parameters of breast tissues. Spectroscopic parameters extracted from frequency-dependent $|G^*(\omega)|$, $G'(\omega)$, $G''(\omega)$, and $\alpha(\omega)$ spectra measured by WB-SHEAR. Unless otherwise specified, values of G and ω are reported in units of kPa and rad/s, respectively. Values indicate mean±standard deviation of N=3 measurements (3 independent locations in each specimen).

	Optical properties (mm^{-1})	Particle radius, *a* (µm)
**Fibrin construct** (Fig. 1)	μ_s' 0.6	0.18*
**Whole blood clot** (Fig. 2)	μ_s' 2.1, μ_a 0.1	2.88†
**Breast tissue** (Fig. 3)	μ_s' 1.5–3.2†	0.1‡
**Bone** (Fig. 4)	μ_s' 4.8 (cortical)	0.012§
	μ_s' 3.5 (trabecular)	

*Experimentally estimated via azimuth DRP obtained with parallel polarization (*46*).

†Based on the hydrodynamic radius of RBC (*91*), assuming RBCs as the primary contributor of light scattering in whole blood.

‡Average scattering particle radii experimentally estimated via the ratio of speckle decorrelation rate of the parallel versus perpendicularly polarized component in *N*=23 breast tissue specimens (*53*).

§Based on the crystal size of bone minerals (*92*), assuming mineral contents as the primary contributor of light scattering in bone.

Table S3. Optical properties and scattering particle size of fibrin constructs, whole blood clots, breast tissues, and bones. Estimated material parameters used in the reconstruction of frequency-dependent $G^*(\omega)$ as detailed in Methods. μ_s': reduced scattering coefficient, μ_a: absorption coefficient, a: sphere-equivalent hydrodynamic radius.

Sample	Histological region	Criteria
**Benign breast tissue**	Fibrous stroma (Fig. 5C)	$F_{HE} > 0.5$ or $F_{PSR} > 0.5$
	Fibro-fatty matrix (Fig. 5D)	$A_{HE} > 0.75$ and $F_{PSR} < 0.25$
**Breast tumor**	Fibrous stroma (Fig. 5G)	$F_{PSR} > 0.5$
	Cellular epithelium (Fig. 5H)	$F_{HE} < 0.2$ and $F_{PSR} < 0.2$

Table S4. Histologiocal identification of WB-SHEAR measurements corresponding to fibrous stroma, cellular epithelium, and fibro-fatty matrix. Criteria based on fraction of pixels in the histological masks obtained from the digitized H&E and PSR images (see Methods). The solid curves and shaded areas in Fig. 5, C, D, G, and H correspond to median±median absolute difference of all WB-SHEAR spectra from the measurement locations that satify the listed criteria. F_{HE}: fibrous stroma from H&E image, A_{HE}: adipose from H&E image, F_{PSR}: fibrous stroma from PSR image.

Parameters	Benign breast tissue		Breast tumor	
	**Fibrous** N=45 (Fig. 5C)	**Fibro-fatty** N=43 (Fig. 5D)	**Fibrous** N=46 (Fig. 5G)	**Cellular** N=5 (Fig. 5H)
Regime I				
ω_T	27±9	23±8	20±8	26±4
Regime II				
ω^0_{II}	170±80*	300±100*	120±20	130±20
G^0_{II}	2.2±1.5†	1.1±0.6†	0.7±0.3§	0.5±0.2
Regime III				
γ_{IIIa}	0.3±0.1‡	0.4±0.3	0.3±0.1‖	0.41±0.06
α_{IIIa}	-	0.2±0.1	0.21±0.04	0.38±0.03
$\omega_{T,III}$	-	-	-	-
ω_γ	1500±900	-	-	700±100
γ_{IIIb}	0.5±0.2‡	-	-	0.53±0.09
α_{IIIb}	0.2±0.1	-	-	0.40±0.04¶
Regime IV				
$\omega^0_{IV}\times10^3$	-	-	3.0±0.8	-
G^0_{IV}	-	-	1.3±0.7§	-
Regime V				
γ_V	-	-	0.5±0.1‖	-
α_V	-	-	0.22±0.06¶	-

Mann-Whitney U-test comparing two spectroscopic paramters indicated by symbols: *P=0.007, †P=0.009, ‡P=0.2, §P=0.006, ‖P=0.00001, ¶P=0.008.

Table S5. Wideband spectroscopic parameters of regions of fibrous stroma, cellular epithelium, and fibro-fatty matrix in benign breast tissue and breast tumor. Spectroscopic parameters extracted from frequency-dependent $|G^*(\omega)|$, $G'(\omega)$, $G''(\omega)$, and $\alpha(\omega)$ spectra measured by WB-SHEAR. Unless otherwise specified, values of G and ω are reported in units of kPa and rad/s, respectively. Values indicate median±median absolute difference of N=45 (Fig. 5C), 43 (Fig. 5D), 46 (Fig. 5G) and 5 (Fig. 5H) measurement locations in each specimen.

Movie S1. Micromechanical mapping of the wideband viscoelastic spectra in benign breast tissue. Right: Frequency-dependent maps of $G'(\omega)$ (top) and $G''(\omega)$ (bottom). Scale bar: 50 μm. Left: Frequency-dependent $|G^*(\omega)|$ (black), $G'(\omega)$ (blue), and $G''(\omega)$ (red) spectra at the locations indicated by + (top) and ○ (bottom) on the maps.

Movie S2. Micromechanical mapping of the wideband viscoelastic spectra in breast tumor. Right: Frequency-dependent maps of $G'(\omega)$ (top) and $G''(\omega)$ (bottom). Scale bar: 50 μm. Left: Frequency-dependent $|G^*(\omega)|$ (black), $G'(\omega)$ (blue), and $G''(\omega)$ (red) spectra at the locations indicated by + (top) and ○ (bottom) on the maps.

REFERENCES AND NOTES

1. D. E. Ingber, Mechanobiology and diseases of mechanotransduction. *Ann. Med.* **35**, 564–577 (2003).

2. D. E. Discher, P. Janmey, Y. L. Wang, Tissue cells feel and respond to the stiffness of their substrate. *Science* **310**, 1139–1143 (2005).

3. M. C. Lampi, C. A. Reinhart-King, Targeting extracellular matrix stiffness to attenuate disease: From molecular mechanisms to clinical trials. *Sci. Transl. Med.* **10**, eaao0475 (2018).

4. D. Wirtz, K. Konstantopoulos, P. C. Searson, The physics of cancer: The role of physical interactions and mechanical forces in metastasis. *Nat. Rev. Cancer* **11**, 512–522 (2011).

5. H. Yu, J. K. Mouw, V. M. Weaver, Forcing form and function: Biomechanical regulation of tumor evolution. *Trends Cell Biol.* **21**, 47–56 (2011).

6. H. T. Nia, L. L. Munn, R. K. Jain, Physical traits of cancer. *Science* **370**, eaaz0868 (2020).

7. E. Nader, S. Skinner, M. Romana, R. Fort, N. Lemonne, N. Guillot, A. Gauthier, S. Antoine-Jonville, C. Renoux, M. D. Hardy-Dessources, E. Stauffer, P. Joly, Y. Bertrand, P. Connes, Blood rheology: Key parameters, impact on blood flow, role in sickle cell disease and effects of exercise. *Front. Physiol.* **10**, 1329 (2019).

8. Y. Qiu, D. R. Myers, W. A. Lam, The biophysics and mechanics of blood from a materials perspective. *Nat. Rev. Mater.* **4**, 294–311 (2019).

9. P. Doradla, K. Otsuka, A. Nadkarni, M. Villiger, A. Karanasos, L. J. C. van Zandvoort, J. Dijkstra, F. Zijlstra, G. van Soest, J. Daemen, E. Regar, B. E. Bouma, S. K. Nadkarni, Biomechanical stress profiling of coronary atherosclerosis: Identifying a multifactorial metric to evaluate plaque rupture risk. *JACC Cardiovasc. Imaging* **13**, 804–816 (2020).

10. K. Driscoll, A. D. Cruz, J. T. Butcher, Inflammatory and biomechanical drivers of endothelial-interstitial interactions in calcific aortic valve disease. *Circ. Res.* **128**, 1344–1370 (2021).

11. A. Seneviratne, M. Hulsmans, P. Holvoet, C. Monaco, Biomechanical factors and macrophages in plaque stability. *Cardiovasc. Res.* **99**, 284–293 (2013).

12. C. Huang, R. Ogawa, Fibroproliferative disorders and their mechanobiology. *Connect. Tissue Res.* **53**, 187–196 (2012).

13. E. F. Morgan, G. U. Unnikrisnan, A. I. Hussein, Bone mechanical properties in healthy and diseased states. *Annu. Rev. Biomed. Eng.* **20**, 119–143 (2018).

14. M. J. Paszek, N. Zahir, K. R. Johnson, J. N. Lakins, G. I. Rozenberg, A. Gefen, C. A. Reinhart-King, S. S. Margulies, M. Dembo, D. Boettiger, D. A. Hammer, V. M. Weaver, Tensional homeostasis and the malignant phenotype. *Cancer Cell* **8**, 241–254 (2005).

15. K. R. Levental, H. Yu, L. Kass, J. N. Lakins, M. Egeblad, J. T. Erler, S. F. Fong, K. Csiszar, A. Giaccia, W. Weninger, M. Yamauchi, D. L. Gasser, V. M. Weaver, Matrix crosslinking forces tumor progression by enhancing integrin signaling. *Cell* **139**, 891–906 (2009).

16. I. Acerbi, L. Cassereau, I. Dean, Q. Shi, A. Au, C. Park, Y. Y. Chen, J. Liphardt, E. S. Hwang, V. M. Weaver, Human breast cancer invasion and aggression correlates with ECM stiffening and immune cell infiltration. *Integr. Biol.* **7**, 1120–1134 (2015).

17. S. P. Carey, Z. E. Goldblatt, K. E. Martin, B. Romero, R. M. Williams, C. A. Reinhart-King, Local extracellular matrix alignment directs cellular protrusion dynamics and migration through Rac1 and FAK. *Integr. Biol.* **8**, 821–835 (2016).

18. A. Labernadie, T. Kato, A. Brugues, X. Serra-Picamal, S. Derzsi, E. Arwert, A. Weston, V. Gonzalez-Tarrago, A. Elosegui-Artola, L. Albertazzi, J. Alcaraz, P. Roca-Cusachs, E. Sahai, X. Trepat, A mechanically active heterotypic E-cadherin/N-cadherin adhesion enables fibroblasts to drive cancer cell invasion. *Nat. Cell Biol.* **19**, 224–237 (2017).

19. C. J. Hernandez, M. C. van der Meulen, Understanding bone strength is not enough. *J. Bone Miner. Res.* **32**, 1157–1162 (2017).

20. S. Vennin, A. Desyatova, J. A. Turner, P. A. Watson, J. M. Lappe, R. R. Recker, M. P. Akhter, Intrinsic material property differences in bone tissue from patients suffering low-trauma osteoporotic fractures, compared to matched non-fracturing women. *Bone* **97**, 233–242 (2017).

21. J. W. Weisel, Structure of fibrin: Impact on clot stability. *J. Thromb. Haemost.* **5**, 116–124 (2007).

22. D. Whiting, J. A. DiNardo, TEG and ROTEM: Technology and clinical applications. *Am. J. Hematol.* **89**, 228–232 (2014).

23. O. Chaudhuri, J. Cooper-White, P. A. Janmey, D. J. Mooney, V. B. Shenoy, Effects of extracellular matrix viscoelasticity on cellular behaviour. *Nature* **584**, 535–546 (2020).

24. Y. Abidine, A. Giannetti, J. Revilloud, V. M. Laurent, C. Verdier, Viscoelastic properties in cancer: From cells to spheroids. *Cells* **10**, 1704 (2021).

25. B. Corominas-Murtra, N. I. Petridou, Viscoelastic networks: Forming cells and tissues. *Front. Phys.* **9**, 666916 (2021).

26. A. R. Cameron, J. E. Frith, J. J. Cooper-White, The influence of substrate creep on mesenchymal stem cell behaviour and phenotype. *Biomaterials* **32**, 5979–5993 (2011).

27. O. Chaudhuri, L. Gu, D. Klumpers, M. Darnell, S. A. Bencherif, J. C. Weaver, N. Huebsch, H. P. Lee, E. Lippens, G. N. Duda, D. J. Mooney, Hydrogels with tunable stress relaxation regulate stem cell fate and activity. *Nat. Mater.* **15**, 326–334 (2016).

28. E. E. Charrier, K. Pogoda, R. Li, R. G. Wells, P. A. Janmey, Elasticity-dependent response of malignant cells to viscous dissipation. *Biomech. Model. Mechanobiol.* **20**, 145–154 (2021).

29. P. U. Shirke, H. Goswami, V. Kumar, D. Shah, S. Beri, S. Das, J. Bellare, S. Mayor, K. V. Venkatesh, J. R. Seth, A. Majumder, "Viscotaxis"- directed migration of mesenchymal stem cells in response to loss modulus gradient. *Acta Biomater.* **135**, 356–367 (2021).

30. A. Elosegui-Artola, A. Gupta, A. J. Najibi, B. R. Seo, R. Garry, C. M. Tringides, I. de Lázaro, M. Darnell, W. Gu, Q. Zhou, D. A. Weitz, L. Mahadevan, D. J. Mooney, Matrix viscoelasticity controls spatiotemporal tissue organization. *Nat. Mater.* **22**, 117–127 (2023).

31. B. A. Krajina, B. L. LeSavage, J. G. Roth, A. W. Zhu, P. C. Cai, A. J. Spakowitz, S. C. Heilshorn, Microrheology reveals simultaneous cell-mediated matrix stiffening and fluidization that underlie breast cancer invasion. *Sci. Adv.* **7**, eabe1969 (2021).

32. N. Leartprapun, S. G. Adie, Recent advances in optical elastography and emerging opportunities in the basic sciences and translational medicine [Invited]. *Biomed. Opt. Express* **14**, 208–248 (2023).

33. P. H. Wu, D. R. Aroush, A. Asnacios, W. C. Chen, M. E. Dokukin, B. L. Doss, P. Durand-Smet, A. Ekpenyong, J. Guck, N. V. Guz, P. A. Janmey, J. S. H. Lee, N. M. Moore, A. Ott, Y. C. Poh, R. Ros, M. Sander, I. Sokolov, J. R. Staunton, N. Wang, G. Whyte, D. Wirtz, A comparison of methods to assess cell mechanical properties. *Nat. Methods* **15**, 491–498 (2018).

34. D. Wirtz, Particle-tracking microrheology of living cells: Principles and applications. *Annu. Rev. Biophys.* **38**, 301–326 (2009).

35. T. G. Mason, D. A. Weitz, Optical measurements of frequency-dependent linear viscoelastic moduli of complex fluids. *Phys. Rev. Lett.* **74**, 1250–1253 (1995).

36. B. A. Krajina, C. Tropini, A. Zhu, P. DiGiacomo, J. L. Sonnenburg, S. C. Heilshorn, A. J. Spakowitz, Dynamic light scattering microrheology reveals multiscale viscoelasticity of polymer gels and precious biological materials. *ACS Cent. Sci.* **3**, 1294–1303 (2017).

37. T. G. Mason, H. Gang, D. A. Weitz, Diffusing-wave-spectroscopy measurements of viscoelasticity of complex fluids. *J. Opt. Soc. Am. A* **14**, 139–149 (1997).

38. R. R. Brau, J. M. Ferrer, H. Lee, C. E. Castro, B. K. Tam, P. B. Tarsa, P. Matsudaira, M. C. Boyce, R. D. Kamm, M. J. Lang, Passive and active microrheology with optical tweezers. *J. Opt. A: Pure Appl. Opt.* **9**, S103–S112 (2007).

39. Y. Lin, N. Leartprapun, S. G. Adie, Spectroscopic photonic force optical coherence elastography. *Opt. Lett.* **44**, 4897–4900 (2019).

40. J. R. Staunton, W. Y. So, C. D. Paul, K. Tanner, High-frequency microrheology in 3D reveals mismatch between cytoskeletal and extracellular matrix mechanics. *Proc. Natl. Acad. Sci. U.S.A.* **116**, 14448–14455 (2019).

41. J. A. Mulligan, G. R. Untracht, S. Chandrasekaran, C. N. Brown, S. G. Adie, Emerging approaches for high-resolution imaging of tissue biomechanics with optical coherence elastography. *IEEE J. Sel. Top. Quantum Electron.* **22**, 246–265 (2016).

42. K. V. Larin, D. D. Sampson, Optical coherence elastography - OCT at work in tissue biomechanics [Invited]. *Biomed. Opt. Express* **8**, 1172–1202 (2017).

43. R. Prevedel, A. Diz-Munoz, G. Ruocco, G. Antonacci, Brillouin microscopy: An emerging tool for mechanobiology. *Nat. Methods* **16**, 969–977 (2019).

44. Z. Hajjarian, S. K. Nadkarni, Evaluation and correction for optical scattering variations in laser speckle rheology of biological fluids. *PLOS ONE* **8**, e65014 (2013).

45. Z. Hajjarian, S. K. Nadkarni, Correction of optical absorption and scattering variations in laser speckle rheology measurements. *Opt. Express* **22**, 6349–6361 (2014).

46. Z. Hajjarian, S. K. Nadkarni, Estimation of particle size variations for laser speckle rheology of materials. *Opt. Lett.* **40**, 764–767 (2015).

47. Z. Hajjarian, H. T. Nia, S. Ahn, A. J. Grodzinsky, R. K. Jain, S. K. Nadkarni, Laser speckle rheology for evaluating the viscoelastic properties of hydrogel scaffolds. *Sci. Rep.* **6**, 37949 (2016).

48. Z. Hajjarian, M. M. Tripathi, S. K. Nadkarni, Optical thromboelastography to evaluate whole blood coagulation. *J. Biophotonics* **8**, 372–381 (2015).

49. D. M. Tshikudi, M. M. Tripathi, Z. Hajjarian, E. M. Van Cott, S. K. Nadkarni, Optical sensing of anticoagulation status: Towards point-of-care coagulation testing. *PLOS ONE* **12**, e0182491 (2017).

50. M. M. Tripathi, D. M. Tshikudi, Z. Hajjarian, D. C. Hack, E. M. Van Cott, S. K. Nadkarni, Comprehensive blood coagulation profiling in patients using iCoagLab: Comparison against thromboelastography. *Thromb. Haemost.* **120**, 1116–1127 (2020).

51. Z. Hajjarian, J. D. Toussaint, J. L. Guerrero, S. K. Nadkarni, In-vivo mechanical characterization of coronary atherosclerotic plaques in living swine using intravascular laser speckle imaging. *Biomed. Opt. Express* **12**, 2064–2078 (2021).

52. S. K. Nadkarni, B. E. Bouma, T. Helg, R. Chan, E. Halpern, A. Chau, M. S. Minsky, J. T. Motz, S. L. Houser, G. J. Tearney, Characterization of atherosclerotic plaques by laser speckle imaging. *Circulation* **112**, 885–892 (2005).

53. Z. Hajjarian, E. F. Brachtel, D. M. Tshikudi, S. K. Nadkarni, Mapping mechanical properties of the tumor microenvironment by laser speckle rheological microscopy. *Cancer Res.* **81**, 4874–4885 (2021).

54. T. G. Mason, K. Ganesan, J. H. van Zanten, D. Wirtz, S. C. Kuo, Particle tracking microrheology of complex fluids. *Phys. Rev. Lett.* **79**, 3282–3285 (1997).

55. T. G. Mason, Estimating the viscoelastic moduli of complex fluids using the generalized Stokes-Einstein equation. *Rheol. Acta* **39**, 371–378 (2000).

56. B. R. Dasgupta, S. Y. Tee, J. C. Crocker, B. J. Frisken, D. A. Weitz, Microrheology of polyethylene oxide using diffusing wave spectroscopy and single scattering. *Phys. Rev. E* **65**, 051505 (2002).

57. M. Doi, S. F. Edwards, *The Theory of Polymer Dynamics*. (Clarendon Press, 1988).

58. D. C. Morse, Viscoelasticity of concentrated isotropic solutions of semiflexible polymers. 2. Linear response. *Macromolecules* **31**, 7044–7067 (1998).

59. M. A. Kotlarchyk, S. G. Shreim, M. B. Alvarez-Elizondo, L. C. Estrada, R. Singh, L. Valdevit, E. Kniazeva, E. Gratton, A. J. Putnam, E. L. Botvinick, Concentration independent modulation of local micromechanics in a fibrin gel. *PLOS ONE* **6**, e20201 (2011).

60. A. R. Wufsus, K. Rana, A. Brown, J. R. Dorgan, M. W. Liberatore, K. B. Neeves, Elastic behavior and platelet retraction in low- and high-density fibrin gels. *Biophys. J.* **108**, 173–183 (2015).

61. N. A. Kurniawan, B. E. Vos, A. Biebricher, G. J. Wuite, E. J. Peterman, G. H. Koenderink, Fibrin networks support recurring mechanical loads by adapting their structure across multiple scales. *Biophys. J.* **111**, 1026–1034 (2016).

62. E. Sarmiento-Gomez, I. Santamaría-Holek, R. Castillo, Mean-square displacement of particles in slightly interconnected polymer networks. *J. Phys. Chem. B* **118**, 1146–1158 (2014).

63. G. H. Koenderink, M. Atakhorrami, F. C. MacKintosh, C. F. Schmidt, High-frequency stress relaxation in semiflexible polymer solutions and networks. *Phys. Rev. Lett.* **96**, 138307 (2006).

64. L. Deng, X. Trepat, J. P. Butler, E. Millet, K. G. Morgan, D. A. Weitz, J. J. Fredberg, Fast and slow dynamics of the cytoskeleton. *Nat. Mater.* **5**, 636–640 (2006).

65. M. Shayegan, N. R. Forde, Microrheological characterization of collagen systems: From molecular solutions to fibrillar gels. *PLOS ONE* **8**, e70590 (2013).

66. A. Rigato, A. Miyagi, S. Scheuring, F. Rico, High-frequency microrheology reveals cytoskeleton dynamics in living cells. *Nat. Phys.* **13**, 771–775 (2017).

67. E. A. Ryan, L. F. Mockros, J. W. Weisel, L. Lorand, Structural origins of fibrin clot rheology. *Biophys. J.* **77**, 2813–2826 (1999).

68. N. A. Kurniawan, T. H. S. van Kempen, S. Sonneveld, T. T. Rosalina, B. E. Vos, K. A. Jansen, G. W. M. Peters, F. N. van de Vosse, G. H. Koenderink, Buffers strongly modulate fibrin self-assembly into fibrous networks. *Langmuir* **33**, 6342–6352 (2017).

69. Z. Zeng, M. Fagnon, T. N. Chakravarthula, N. J. Alves, Fibrin clot formation under diverse clotting conditions: Comparing turbidimetry and thromboelastography. *Thromb. Res.* **187**, 48–55 (2020).

70. E. Blauth, H. Kubitschke, P. Gottheil, S. Grosser, J. A. Käs, Jamming in embryogenesis and cancer progression. *Front. Phys.* **9**, 666709 (2021).

71. O. Ilina, P. G. Gritsenko, S. Syga, J. Lippoldt, C. A. M. La Porta, O. Chepizhko, S. Grosser, M. Vullings, G. Bakker, J. Starruß, P. Bult, S. Zapperi, J. A. Käs, A. Deutsch, P. Friedl, Cell–cell adhesion and 3D matrix confinement determine jamming transitions in breast cancer invasion. *Nat. Cell Biol.* **22**, 1103–1115 (2020).

72. W. M. Allen, K. M. Kennedy, Q. Fang, L. Chin, A. Curatolo, L. Watts, R. Zilkens, S. L. Chin, B. F. Dessauvagie, B. Latham, C. M. Saunders, B. F. Kennedy, Wide-field quantitative micro-elastography of human breast tissue. *Biomed. Opt. Express* **9**, 1082–1096 (2018).

73. E. V. Gubarkova, A. A. Sovetsky, V. Y. Zaitsev, A. L. Matveyev, D. A. Vorontsov, M. A. Sirotkina, L. A. Matveev, A. A. Plekhanov, N. P. Pavlova, S. S. Kuznetsov, A. Y. Vorontsov, E. V. Zagaynova, N. D. Gladkova, OCT-elastography-based optical biopsy for breast cancer delineation and express assessment of morphological/molecular subtypes. *Biomed. Opt. Express* **10**, 2244–2263 (2019).

74. E. V. Gubarkova, E. B. Kiseleva, M. A. Sirotkina, D. A. Vorontsov, K. A. Achkasova, S. S. Kuznetsov, K. S. Yashin, A. L. Matveyev, A. A. Sovetsky, L. A. Matveev, A. A. Plekhanov, A. Y. Vorontsov, V. Y. Zaitsev, N. D. Gladkova, Diagnostic accuracy of cross-polarization OCT and OCT-elastography for differentiation of breast cancer subtypes: Comparative study. *Diagnostics (Basel)* **10**, 994 (2020).

75. M. Granke, M. D. Does, J. S. Nyman, The role of water compartments in the material properties of cortical bone. *Calcif. Tissue Int.* **97**, 292–307 (2015).

76. R. K. Surowiec, M. R. Allen, J. M. Wallace, Bone hydration: How we can evaluate it, what can it tell us, and is it an effective therapeutic target? *Bone Rep.* **16**, 101161 (2022).

77. I. K. Piechocka, R. G. Bacabac, M. Potters, F. C. MacKintosh, G. H. Koenderink, Structural hierarchy governs fibrin gel mechanics. *Biophys. J.* **98**, 2281–2289 (2010).

78. B. Gong, X. Wei, J. Qian, Y. Lin, Modeling and simulations of the dynamic behaviors of actin-based cytoskeletal networks. *ACS Biomater Sci. Eng.* **5**, 3720–3734 (2019).

79. L. G. Rizzi, Physics-based computational approaches to compute the viscoelasticity of semiflexible filamentous biomaterials. *Front. Phys.* **10**, 893613 (2022).

80. M. Bellour, M. Skouri, J. P. Munch, P. Hébraud, Brownian motion of particles embedded in a solution of giant micelles. *Eur. Phys. J. E* **8**, 431–436 (2002).

81. J. Galvan-Miyoshi, J. Delgado, R. Castillo, Diffusing wave spectroscopy in Maxwellian fluids. *Eur. Phys. J. E* **26**, 369–377 (2008).

82. J. Scholler, K. Groux, O. Goureau, J. A. Sahel, M. Fink, S. Reichman, C. Boccara, K. Grieve, Dynamic full-field optical coherence tomography: 3D live-imaging of retinal organoids. *Light Sci. Appl.* **9**, 140 (2020).

83. H. M. Leung, M. L. Wang, H. Osman, E. Abouei, C. MacAulay, M. Follen, A. Gardecki, G. J. Tearney, Imaging intracellular motion with dynamic micro-optical coherence tomography. *Biomed. Opt. Express* **11**, 2768–2778 (2020).

84. M. Keating, A. Kurup, M. Alvarez-Elizondo, A. J. Levine, E. Botvinick, Spatial distributions of pericellular stiffness in natural extracellular matrices are dependent on cell-mediated proteolysis and contractility. *Acta Biomater.* **57**, 304–312 (2017).

85. S. E. Leggett, M. Patel, T. M. Valentin, L. Gamboa, A. S. Khoo, E. K. Williams, C. Franck, I. Y. Wong, Mechanophenotyping of 3D multicellular clusters using displacement arrays of rendered tractions. *Proc. Natl. Acad. Sci. U.S.A.* **117**, 5655–5663 (2020).

86. J. A. Mulligan, L. Ling, N. Leartprapun, C. Fischbach, S. G. Adie, Computational 4D-OCM for label-free imaging of collective cell invasion and force-mediated deformations in collagen. *Sci. Rep.* **11**, 2814 (2021).

87. Y. Lin, N. Leartprapun, J. C. Luo, S. G. Adie, Light-sheet photonic force optical coherence elastography for high-throughput quantitative 3D micromechanical imaging. *Nat. Commun.* **13**, 3495 (2022).

88. S. Nadkarni, A. Bilenca, B. E. Bouma, G. J. Tearney, Measurement of fibrous cap thickness in atherosclerotic plaques by spatiotemporal analysis of laser speckle images. *J. Biomed. Opt.* **11**, 021006 (2006).

89. R. Maltais-Tariant, C. Boudoux, N. Uribe-Patarroyo, Real-time co-localized OCT surveillance of laser therapy using motion corrected speckle decorrelation. *Biomed. Opt. Express* **11**, 2925–2950 (2020).

90. M. T. Valentine, Z. E. Perlman, M. L. Gardel, J. H. Shin, P. Matsudaira, T. J. Mitchison, D. A. Weitz, Colloid surface chemistry critically affects multiple particle tracking measurements of biomaterials. *Biophys. J.* **86**, 4004–4014 (2004).

91. M. Friebel, A. Roggan, G. Müller, M. Meinke, Determination of optical properties of human blood in the spectral range 250 to 1100 nm using Monte Carlo simulations with hematocrit-dependent effective scattering phase functions. *J. Biomed. Opt.* **11**, 034021 (2006).

92. M. J. Turunen, J. D. Kaspersen, U. Olsson, M. Guizar-Sicairos, M. Bech, F. Schaff, M. Tägil, J. S. Jurvelin, H. Isaksson, Bone mineral crystal size and organization vary across mature rat bone cortex. *J. Struct. Biol.* **195**, 337–344 (2016).
